# Infection and genotype remodel the entire soybean transcriptome

**DOI:** 10.1186/1471-2164-10-49

**Published:** 2009-01-26

**Authors:** Lecong Zhou, Santiago X Mideros, Lei Bao, Regina Hanlon, Felipe D Arredondo, Sucheta Tripathy, Konstantinos Krampis, Adam Jerauld, Clive Evans, Steven K St Martin, MA Saghai Maroof, Ina Hoeschele, Anne E Dorrance, Brett M Tyler

**Affiliations:** 1 Virginia Bioinformatics Institute, Virginia Polytechnic Institute and State University, Blacksburg, VA 24061, USA; 2 Department of Crop and Soil Environmental Sciences, Virginia Polytechnic Institute and State University, Blacksburg, VA 24061, USA; 3 Department of Statistics, Virginia Polytechnic Institute and State University, Blacksburg, VA 24061, USA; 4 Department of Plant Pathology, The Ohio State University OARDC, Wooster, OH 44691, USA; 5 Department of Horticulture and Crop Science, The Ohio State University, Columbus, OH 43210, USA

## Abstract

**Background:**

High throughput methods, such as high density oligonucleotide microarray measurements of mRNA levels, are popular and critical to genome scale analysis and systems biology. However understanding the results of these analyses and in particular understanding the very wide range of levels of transcriptional changes observed is still a significant challenge. Many researchers still use an arbitrary cut off such as two-fold in order to identify changes that may be biologically significant. We have used a very large-scale microarray experiment involving 72 biological replicates to analyze the response of soybean plants to infection by the pathogen *Phytophthora sojae *and to analyze transcriptional modulation as a result of genotypic variation.

**Results:**

With the unprecedented level of statistical sensitivity provided by the high degree of replication, we show unambiguously that almost the entire plant genome (97 to 99% of all detectable genes) undergoes transcriptional modulation in response to infection and genetic variation. The majority of the transcriptional differences are less than two-fold in magnitude. We show that low amplitude modulation of gene expression (less than two-fold changes) is highly statistically significant and consistent across biological replicates, even for modulations of less than 20%. Our results are consistent through two different normalization methods and two different statistical analysis procedures.

**Conclusion:**

Our findings demonstrate that the entire plant genome undergoes transcriptional modulation in response to infection and genetic variation. The pervasive low-magnitude remodeling of the transcriptome may be an integral component of physiological adaptation in soybean, and in all eukaryotes.

## Background

How many genes are truly involved in the response of organism to a challenge such as pathogen infection, and what are the roles of those genes? Global assays of gene expression, for example by microarray analysis, are typically conducted to test the hypothesis that a small, defined set of genes are responsible for an organism's response to some challenge. Gene expression changes below a certain threshold (commonly 2 fold) are often disregarded as being irrelevant and/or unreliable. A major challenge in evaluating the importance of low magnitude transcriptional changes is that the level of replication used in a typical microarray experiment is insufficient to detect small changes given the technical and biological variability in the system. Although several methods appear to be promising for precise quantification of gene expression, it remains uncertain what constitutes a significant change in response to treatments [1,2].

High-density oligonucleotide arrays such as Affymetrix GeneChips can detect up to 90% of all the mRNAs in a transcriptome [3-5]. For example, nearly 90% of all yeast mRNAs could be detected in cells grown under both rich and minimal media growth conditions, with approximately 50% being present at average levels between 0.1 and 1 copy per cell [3]. Of the 31,000 genes on Affymetrix Rat Genomic 230 2.0 GeneChip microarrays, 18,200 (58.7%) could be detected in growing rat bone [5]. In a study with human abdominal aortic aneurysms, of the 18,057 genes common to Affymetrix and Illumina arrays, 11,542 (64%) were expressed in either aneurysmal or normal abdominal aorta [6]. Approximately 26,500 of the soybean genes (70%) on the Affymetrix GeneChip could be detected in soybean cyst nematode (SCN)-colonized root pieces[4].

Markedly varied numbers of genes, from only a few up to several thousands, have been reported to be differentially expressed in response to diverse challenges, depending on the system and the statistical methodology. For instance, of the approximately 6,200 protein-encoding genes in the *Saccharomyces cerevisiae *(yeast) genome, over 1,000 showed significant changes in mRNA levels during sporulation [7]. In rat, 8,002 out of 18,200 expressed genes (44.0%) had a significant change in gene expression during growth, about half up-regulated and half down-regulated [5]. In *Arabidopsis thaliana*, 939 out of approximately 24,000 genes showed a statistically significant response to cold stress, with 655 up-regulated and 284 down-regulated [8].

One of the most profound challenges an organism can suffer is pathogen infection. In a meta-analysis of 32 studies involving 785 transcriptomic experiments with 77 different host-pathogen interactions [9], 5042 human genes showed transcriptional changes in response to at least one challenge, and a cluster of 511 co-regulated genes was identified as representing a common infection response. During infection of the plant *Arabidopsis *by the bacterial pathogen *Pseudomonas syringae*, approximately 2,000 of the approximately 8,000 genes monitored showed significant expression level changes [10]. In soybean, the Affymetrix GeneChip has been used to profile gene expression during infection with soybean rust fungus and soybean cyst nematode (SCN) [4,11-14]. During nematode infection, 429 of 35611 soybean transcripts (which account for 1.2%), while 1850 out of 7430 SCN genes (24.9%) showed expression changes [4].

To identify genes involved in the responses of several soybean genotypes to infection by the oomycete pathogen *Phytophthora sojae*, we conducted a very large-scale microarray experiment using Affymetrix GeneChips. Three soybean genotypes (V71-370, Sloan and VPRIL9) were included within each of the 29 experimental blocks. Replicates of each set of the three genotypes, incubated in the same growth chamber, were harvested at three different times (9 am, 10:30 am, and 12 pm). For each soybean line, approximately 30 seedlings were inoculated on the roots with *P. sojae *and after 5 days, 7.5 mm root segments were collected from immediately above and below the upper margin of the visible lesion (referred to from here as the "Upper" and "Lower" infection courts). From approximately 20 mock-inoculated plants, 15 mm root segments ("Mock") were collected from positions on the roots matching the lesions on the pathogen-inoculated plants. RNA was extracted from each of the samples, labeled and hybridized to Affymetrix soybean GeneChips. This paper reports our analyses of a total of 648 GeneChips with the samples obtained from 24 blocks from which a full set of 27 successful microarray assays were obtained.

## Results

### The entire soybean transcriptome is significantly remodeled by genotypic differences and *Phytophthora *infection

The primary GeneChip data were pre-processed using GC-RMA background correction, quantile normalization and median polish summarization. To identify factors that significantly affected expression of individual genes, Linear Mixed-Model Analysis (LMMA) was used, and three methods to control the False Discovery Rate (FDR) were employed.

LMMA indicated that 89% of the detectable genes (i.e. those with a significant hybridization signal determined by the MAS5 presence-absence test) were significantly affected by infection in both the upper and lower infection courts, even when using the most conservative FDR method (BH-FDR) at a level of 0.0001 (Table 1). With the least conservative method (q-value) at a level of 0.05, 98.3% of the detectable genes were identified as significantly affected. The analysis also identified very large numbers of genes whose transcription level was significantly affected by genotype. For instance, at moderate stringency (TST-adjusted p ≤ 0.01), 25,923 genes (91.4% of all detectable genes) showed significant expression differences among the three soybean lines. Furthermore, 24,669 (87.0%) showed a genotype-sensitive response to infection (significant genotype × infection interaction; TST-adjusted p ≤ 0.01). Genes showing significant responses to infection and/or infection × genotype interaction constituted 98.2% to 99.4% of the detectable transcriptome (27,831 or 28,187 genes with TST-adjusted p ≤ 0.01 or 0.05 respectively). Genes showing significant responses to genotype and/or infection × genotype interaction constituted 97.7% to 99.4% of the detectable transcriptome (27,710 or 28,172 genes with TST-adjusted p ≤ 0.01 or 0.05 respectively). The experiment was also able to identify a large number of genes that responded significantly to the subtle differences in the times of harvest (9 am, 10:30 am, 12 noon) (4,453 genes = 15.7% at TST-adjusted p ≤ 0.01), although as expected this number was smaller than the overall response to infection and genotype.

**Table 1:** Linear mixed-model analysis (LMMA) of soybean gene expression changes under infection by *P. sojae*.

			Fixed factors^a^			
			
FDR^b^		Significant genes^c^	Genotype^d^	Treatment^e^	Time^f^	Genotype × Treatment
0.05	BH	Number	26,262	27,285	5,335	25,100
		Percent	92.6	96.2	18.8	88.5
	TST	Number	27,156	27,732	9,960	26,500
		Percent	95.8	97.8	35.1	93.5
	q-value	Number	27,430	27,872	12,602	27,008
		Percent	96.8	98.3	44.4	95.3

0.01	BH	Number	25,062	26,761	2,965	23,552
		Percent	88.4	94.4	10.5	83.1
	TST	Number	25,923	27,139	4,453	24,669
		Percent	91.4	95.7	15.7	87.0
	q-value	Number	26,337	27,316	5,561	25,206
		Percent	92.9	96.3	19.6	88.9

0.001	BH	Number	23,542	26,015	1,575	21,445
		Percent	83.0	91.8	5.6	75.6
	TST	Number	24,191	26,351	2,025	22,381
		Percent	85.3	92.9	7.1	78.9
	q-value	Number	24,657	26,582	2,488	23,012
		Percent	87.0	93.8	8.8	81.2

0.0001	BH	Number	21,988	25,272	960	19,577
		Percent	77.6	89.1	3.4	69.1
	TST	Number	22,611	25,560	1,173	20,293
		Percent	79.8	90.2	4.1	71.6
	q-value	Number	23,150	25,836	1,381	20,922
		Percent	81.7	91.1	4.9	73.8

^a^Model: y = Genotype + Treatment + Time + Genotype × Treatment + Block + Block × Genotype + Block × Treatment + Block × Time + Block × Genotype × Treatment + Error, where y refers to the log2 scale median polish summarized gene expression values, the main factors (Genotype, Treatment and Time) and the interaction Genotype × Treatment were fixed factors while all the remaining terms were random factors. GC-RMA background correction and quantile normalization were performed prior to median polish summarization. LMMA was carried out using Proc Mixed of SAS v9. ^b^The p-values for each of the fixed factors were computed based on normality assumption and adjusted for multiple testing using BH, TST and q-value methods at levels from 0.0001 to 0.05. ^c^Numbers of detectable genes significant for each factor at the indicated adjusted p value, and percentages of the total number of detectable genes. ^d^Genotypes were V71-370, Sloan and VPRIL9. ^e^Treatments include Upper, Lower, and Mock. ^f^Time refers to sample harvest time window at 9:00 am, 10:30 am, and 12:00 pm.

To verify that our statistical analysis was effectively controlling false discovery, we separated the 24 blocks into two "sub-groups" consisting of odd-numbered and even-numbered blocks respectively. We then included the effect of sub-group in our linear mixed model and used LMMA to identify genes showing significant differences between the sub-groups. Very few genes showed differences under even the most relaxed criteria (45 at TST-FDR adjusted p ≤ 0.05; Table 2), while at moderate criteria (TST-FDR adjusted p ≤ 0.01) none showed differences. Similarly, when the 24 blocks were randomized into 6 sub-groups, each containing 4 blocks, only 20 genes showed significant differences among sub-groups (TST-FDR adjusted p ≤ 0.01). These small numbers of genes that show differences among sub-groups may reflect genes that are unusually sensitive to small variations in conditions used to assay the different biological replicates.

**Table 2:** Differences among sub-groups as revealed by LMMA analyses of data sets preprocessed by GC-RMA (TST-FDR)

FDR^a^	Fixed factors^b^	2 Sub-Groups^c^		6 Sub-Groups^d^	
		
		Number significant^e^	Percentage significant^f^	Number significant^e^	Percentage significant^f^
0.05	Genotype	26,043	91.9	25,975	91.6
	Treatment	27,191	95.9	27,180	95.9
	Time	4,748	16.7	4,735	16.7
	Genotype × Treatment	24,837	87.6	24,774	87.4
	Sub	45	0.2	235	0.8
	Sub × Genotype	458	1.6	238	0.8
	Sub × Treatment	251	0.9	429	1.5
	Sub × Genotype × Treatment	805	2.8	364	1.3

0.01	Genotype	24,808	87.5	24,732	87.2
	Treatment	26,652	94.0	26,645	94.0
	Time	2,692	9.5	2,692	9.5
	Genotype × Treatment	23,275	82.1	23,213	81.9
	Sub	0	0	20	0.1
	Sub × Genotype	68	0.2	42	0.1
	Sub × Treatment	35	0.1	74	0.3
	Sub × Genotype × Treatment	143	0.5	53	0.2

0.001	Genotype	23,268	82.1	23,150	81.7
	Treatment	25,891	91.3	25,881	91.3
	Time	1,468	5.2	1,465	5.2
	Genotype × Treatment	21,141	74.6	21,056	74.3
	Sub	0	0	0	0
	Sub × Genotype	4	0	3	0
	Sub × Treatment	5	0	9	0
	Sub × Genotype × Treatment	13	0	2	0

0.0001	Genotype	21,694	76.5	21,474	75.7
	Treatment	25,128	88.6	25,073	88.4
	Time	895	3.2	894	3.2
	Genotype × Treatment	19,265	68.0	19,131	67.5
	Sub	0	0	0	0
	Sub × Genotype	0	0	0	0
	Sub × Treatment	2	0	0	0
	Sub × Genotype × Treatment	1	0	0	0

0.00001	Genotype	20,105	71	19,845	70
	Treatment	24,310	86	24,264	86
	Time	585	2	583	2
	Genotype × Treatment	17,518	62	17,377	61
	Sub	0	0	0	0
	Sub × Genotype	0	0	0	0
	Sub × Treatment	0	0	0	0
	Sub × Genotype × Treatment	0	0	0	0

^a^False discovery rate (FDR) was controlled the two-stage linear step-up method (TST-FDR). ^b^The fixed factors used in the LMMA model included genotype (V71-370, Sloan and VPRIL9), treatment (Upper, Lower, and Mock), time (9 am, 10:30 am, and 12 pm), sub (sub-group), and the interactions (genotype × treatment, sub × genotype, sub × treatment, and sub × genotype × treatment). ^c ^"2 Sub-Groups" refers to the 12 odd-numbered blocks compared to the 12 even-numbered blocks. ^d ^"6 Sub-Groups" refers to 6 randomly selected groups of blocks (four blocks in each group). ^e^Number of significant genes for a factor determined by LMMA analysis with FDR control. ^f^The number of significant genes as a percentage of the total number of detectable genes.

To verify that our results did not depend on the preprocessing methods (background subtraction and data normalization), we preprocessed the GeneChip data with the Bioconductor MAS5 algorithm and re-analyzed the results by LMMA. For the fixed factors, genotype, treatment, and genotype × treatment interaction, the agreement between the results of the two methods was between 92% and 98% at all levels of significance (Table 3). For the time factor the agreement was less, but still at least 77.0%.

**Table 3:** Comparison of preprocessing data sets by GC-RMA or MAS5 algorithms on the significance of fixed factors as revealed by LMMA analyses

FDR (TST)^a^	Fixed factor^b^	Number significant genes^c^		Number matches^d^	Percentage matches^e^
				
		GC-RMA	MAS5		
0.05	Genotype	27,156	26,651	25,896	97.2
	Treatment	27,732	27,585	27,099	98.2
	Genotype × Treatment	26,500	25,932	24,977	96.3
	Time	9,960	9,038	6,961	77

0.01	Genotype	25,923	25,162	24,024	95.5
	Treatment	27,139	26,864	26,079	97.1
	Genotype × Treatment	24,669	23,643	22,327	94.4
	Time	4,453	3,655	2,941	80.5

0.001	Genotype	24,191	23,161	21,780	94
	Treatment	26,351	25,962	24,873	95.8
	Genotype × Treatment	22,381	20,924	19,507	93.2
	Time	2,025	1,551	1,348	86.9

0.0001	Genotype	22,611	21,270	19,800	93.1
	Treatment	25,560	25,126	23,817	94.8
	Genotype × Treatment	20,293	18,691	17,256	92.3
	Time	1,173	857	768	89.6

^a^False discovery rate (FDR) was controlled using the two-stage linear step-up method (TST). ^b^The fixed factors used in the LMMA model included genotype (V71-370, Sloan and VPRIL9), treatment (Upper, Lower, and Mock), time (9 am, 10:30 am, and 12 pm), and the genotype × treatment interaction. ^c^Number of significant genes for a factor was determined by LMMA analysis with FDR control. ^d^The number of genes called significant following GC-RMA and following MAS5 pre-processing. ^e^The number of matches as a percentage of the minimum of the number of significant genes called following GC-RMA or MAS5 preprocessing.

To independently verify the results of the LMMA analysis, we employed the non-parametric Wilcoxon signed rank test to analyze the significance of infection responses; data from each of the infection chips were paired with the corresponding data from mock inoculation. The genes identified as significant by this method matched those identified by LMMA with an agreement of > 99%, with either method of pre-processing (GC-RMA or MAS5) (Table 4).

**Table 4:** Comparison of significant infection responses identified with SAS Proc Mixed LMMA or the non-parametric Wilcoxon signed rank test.

Data Pre-Processing^a^	Genotype	Infection Response^b^	Wilcoxon^c^		LMMA^d^		Number Matches^g^	Percentage matches^h^
					
			Significant^e^	% sig^f^	Significant	% sig		
GC-RMA	V71-370	Upper vs. Mock	23,016	81.2	20,418	72.0	20,406	99.94
		Lower vs. Mock	23,739	83.7	21,772	76.8	21,749	99.89
	Sloan	Upper vs. Mock	24,369	86	22,758	80.3	22,712	99.8
		Lower vs. Mock	24,872	87.7	23,556	83.1	23,513	99.82
	VPRIL9	Upper vs. Mock	24,851	87.7	23,543	83.0	23,482	99.74
		Lower vs. Mock	25,260	89.1	24,180	85.3	24,087	99.62

MAS5	V71-370	Upper vs. Mock	22,300	78.7	19,486	68.7	19,478	99.96
		Lower vs. Mock	23,406	82.6	21,772	74.8	21,189	99.96
	Sloan	Upper vs. Mock	23,984	84.6	22,758	78.5	22,208	99.82
		Lower vs. Mock	24,578	86.7	23,556	81.7	23,153	99.94
	VPRIL9	Upper vs. Mock	24,714	87.2	23,543	82.5	23,366	99.91
		Lower vs. Mock	25,144	88.7	24,180	85.4	24,165	99.81

^a^The data used for the analyses were either preprocessed using GC-RMA or using MAS5 algorithm as described in the Methods. ^b^Infection responses relative to mock-inoculated tissue were evaluated for the upper infection court (Upper vs. Mock) and the lower infection court (Lower vs. Mock). ^c^Wilcoxon signed ranks test implemented by the wilcox.test function of R package *stats *version 2.6.0. ^d^Linear mixed model analysis performed in SAS Proc Mixed. Model: y = Genotype + Treatment + Time + Genotype × Treatment + Block + Block × Genotype + Block × Treatment + Block × Time + Block × Genotype × Treatment + Error, where y refers to the log2 scale median polish summarized gene expression values, the main factors (Genotype, Treatment and Time) and the interaction Genotype × Treatment were fixed factors while all the remaining terms were random factors. ^e^Total number of significant genes with TST-FDR adjusted p ≤ 0.01. ^f^Percentage of the total number of detectable genes that are significant. ^g^Number of significant genes found with both Wilcoxon and LMMA. ^h^The number of matches found with both methods, as a percentage of the total number of significant genes found by Wilcoxon or LMMA, whichever was smaller.

### Transcriptional changes display both consistency and fluctuation

To assess to what extent the transcriptional changes we observed were biologically reproducible, we compared our results to those obtained from a pilot experiment carried out two years previously with cultivars V71-370 and Sloan. That pilot experiment had a near identical design except that all plants were harvested at one time during the morning. Also, the upper and lower infection courts were harvested together. To enable comparison with the 72 sample experiment, in which the upper and lower infection courts were harvested separately, the results from the upper and lower courts were averaged. The overall correlation in the expression changes measured for all genes by microarray analysis in the two experiments was very high (R^2 ^= 0.85) indicating a high level of reproducibility. Even genes showing less than two-fold perturbations by infection showed excellent consistency between the two experiments. For example, in V71-370, Gma.3473.1.S1_at (small heat shock protein) was down-regulated 1.2 fold and 1.7 fold in the two experiments, and in Sloan down-regulated 1.7 fold and 2.2 fold. As another example, Gma.6640.1.S1_at (secretory peroxidase) was up-regulated 1.46 fold and 1.07 fold in V71-370, and down-regulated 1.08 fold and 1.16 fold in Sloan. In a third example, GmaAffx.60616.1.S1_at (Hcr9-OR2A) was down-regulated 1.5 fold and 1.35 fold in V71-370, and down-regulated 1.47 fold and 1.8 fold in Sloan. Only one measurement showed a statistically significant difference between the two experiments (GmaAffx.18905.1.S1 cell death associated protein: in Sloan; FDR adjusted p < 0.05). This agreement is remarkable given that this is a two-organism interaction and that two experiments were conducted two years apart with different batches of soybean seed.

During the pilot experiment, transcript levels of 22 genes, including 4 housekeeping genes, were also measured by quantitative real-time PCR (qRT-PCR), using the same RNA preparations as for the microarrays. For the 22 selected genes, the correlation between the microarray measurements from the two experiments was 0.90 (R^2^) and the correlation between the microarray and qRT-PCR measurements was 0.88 (R^2^) (Table 5) [see also Additional file 1]. In most cases, the agreement between the qRT-PCR results and the microarray results was very good. In only two cases (GmaAffx.64261.1.A1_at quinone oxidoreductase and GmaAffx.75675.1.A1_at phenylalanine ammonia-lyase, both in V71-370) were statistically significant differences (FDR-adjusted p < 0.05) observed between the qRT-PCR and the array results; possibly in these cases the two assay methods differed in their sensitivity to the presence of mRNAs from close paralogs and homeologs of the assayed genes.

**Table 5:** Consistency of selected gene expression differences among independent experiments, measured by qRT-PCR and/or microarray.

				Pilot experiment			72-replicate experiment (array)					
				5 day			5 day (average upper and lower versus mock)					
Geno-type^a^	Gene ID^b^	Annotation^c^	Inoc/Mock	qRT	array	Whole^f^	Sub^g ^A	Sub B	Sub C	Sub D	Sub E	Sub F

V71-370	Gma.441.1.S1_at	Ubiquitin (AtRUB1) [HK]	mean^d^	1.17	1.08	1.07	1.08	1.04	1.05	1.21	1.09	-1.01
			s.e.^e^	0.9	0.08	0.03	0.09	0.05	0.08	0.08	0.04	0.09
	
	Gma.16125.1.S1_s_at	Ribosomal protein S27-like [HK]	mean	1.12	1.16	1.15‡	1.17	1.19	1.14	1.16	1.19	1.08
			s.e.	0.15	0.14	0.06	0.10	0.12	0.14	0.16	0.12	0.11
	
	GmaAffx.90824.1.S1_s_at	26S proteasome subunit RPN2a [HK]	mean	-1.25	-1.16	-1.08	-1.00	-1.23	-1.08	-1.12	1.00	-1.02
			s.e.	0.18	0.15	0.04	0.10	0.08	0.09	0.08	0.06	0.08
	
	GmaAffx.90181.1.A1_at	Actin	mean	2.0	1.25	-1.03	1.07	-1.00	-1.11	1.07	-1.11	-1.05
			s.e.	0.8	0.5	0.07	0.17	0.17	0.20	0.17	0.16	0.16
	
	GmaAffx.60616.1.S1_at	Hcr9-OR2A	mean	-1.7	-1.5	-1.35‡	-1.28	-1.31	-1.20	-1.20	-1.37	-1.8
			s.e.	0.49	0.22	0.04	0.10	0.11	0.12	0.09	0.10	0.09
	
	Gma.447.1.S1_at	Defense associated acid phosphatase	mean	-2.8	-1.47	-1.9‡	-1.34	-1.34	-2.9	-1.5	-2.8	-1.9
			s.e.	0.40	0.27	0.05	0.18	0.23	0.06	0.16	0.06	0.11
	
	GmaAffx.64261.1.A1_at	Quinone oxidoreductase	mean	3.6	8.5*‡	5.6‡	5.3	5.4	5.3	7.4	4.4	7.2
			s.e.	0.7	2.7	0.6	1.8	1.3	1.1	1.9	0.9	1.7
	
	Gma.6640.1.S1_at	At5g66390 (secretory peroxidase)	mean	1.5	1.46‡	1.07‡	1.06	1.15	-1.10	1.10	1.11	1.11
			s.e.	0.43	0.10	0.05	0.12	0.11	0.10	0.11	0.09	0.13
	
	GmaAffx.18905.1.S1_at	Cell death associated protein	mean	2.5	2.4‡	2.3‡	2.0	1.9	2.7	2.8	2.4	2.4
			s.e.	0.41	0.7	0.21	0.42	0.42	0.6	0.46	0.46	0.37
	
	Gma.17993.1.S1_at	P21 protein	mean	60	70‡	30‡	29	34	26	40	36	24
			s.e.	13	19	3.7	11	9	7	7	10	9
	
	GmaAffx.75675.1.A1_at	Phenylalanine ammonia-lyase	mean	3.9	7.6*‡	4.4‡	4.4	4.4	4.7	4.0	4.6	4.9
			s.e.	0.41	0.9	0.29	0.9	0.7	0.7	0.46	0.7	0.7
	
	GmaAffx.2799.1.A1_at	NtPRp27-like protein	mean	12	20‡	7.8‡	5.9	7.8	8.0	7.8	9.6	8.3
			s.e.	0.6	5	0.6	1.4	1.4	1.1	0.9	1.2	1.3
	
	Gma.16733.2.S1_at	WRKY4 transcription factor	mean	4.9	6.8‡	2.9‡	3.9	3.5	2.6	3.1	2.9	2.6
			s.e.	0.6	1.7	0.37	1.0	1.2	0.8	0.9	1.1	0.7
	
	GmaAffx.29692.1.S1_at	Chitinase (class II)	mean	1.9	2.9‡	2.0‡	2.4	2.1	1.6	1.9	1.8	2.2
			s.e.	0.47	0.7	0.11	0.34	0.27	0.22	0.20	0.29	0.23
		
	Gma.16911.1.S1_at	Cytochrome P450 family protein	mean	-1.23	1.6	1.24‡	1.07	1.20	1.29	1.21	1.28	1.47
			s.e.	0.26	0.33	0.05	0.11	0.11	0.11	0.11	0.10	0.08
		
	GmaAffx.7209.1.A1_at	UDP-glucose 6-dehydrogenase	mean	-1.02	1.36	-1.02	1.09	1.17	-1.05	-1.03	-1.24	-1.01
			s.e.	0.10	0.21	0.07	0.14	0.22	0.11	0.13	0.13	0.15
		
	GmaAffx.47171.1.A1_at	Putative resistance protein	mean	-1.03	1.43‡	1.6‡	1.35	1.5	1.5	1.6	1.7	1.9
			s.e.	0.12	0.38	0.08	0.17	0.17	0.21	0.16	0.16	0.19
		
	Gma.3473.1.S1_at	Protein, small heat shock	mean	-1.26	-1.20	-1.7‡	1.7	-1.6	-3.4	-1.7	-2.1	-2.3
			s.e.	0.26	0.22	0.06	0.30	0.16	0.08	0.14	0.11	0.08
		
	GmaAffx.90984.1.A1_at	Germin-like protein	mean	14	5.7‡	2.1‡	2.0	2.2	1.9	1.9	2.0	2.4
			s.e.	2.5	2.2	0.16	0.35	0.44	0.31	0.33	0.47	0.39
		
	GmaAffx.32612.1.S1_at	HI4'OMT	mean	18	24‡	14‡	9.6a	8.9a	20b	12a	21b	22b
			s.e.	1.8	6.5	1.5	3.2	2.3	3.3	2.8	4.7	6
		
	GmaAffx.90059.1.S1_at	HcrVf2 protein	mean	7.4	3.7‡	3.0‡	3.2	2.7	2.9	3.2	2.9	3.2
			s.e.	2.0	0.8	0.22	0.5	0.37	0.26	0.41	0.42	0.8
	
	GmaAffx.79817.1.S1_at	SGT1	mean	1.13	1.04	-1.01	1.07	-1.10	1.00	-1.04	-1.11	1.13
			s.e.	0.14	0.07	0.04	0.15	0.08	0.14	0.06	0.05	0.07
	
Sloan	Gma.441.1.S1_at	Ubiquitin (AtRUB1) [HK]	mean^d^	-1.04	1.19	1.12‡	1.07	1.20	1.07	1.23	1.10	1.08
			s.e.^e^	0.10	0.14	0.04	0.09	0.06	0.09	0.12	0.08	0.11
	
	Gma.16125.1.S1_s_at	Ribosomal protein S27-like [HK]	mean	-1.18	1.03	-1.07	1.02	-1.04	-1.14	1.10	-1.17	-1.14
			s.e.	0.5	0.18	0.06	0.08	0.06	0.15	0.22	0.07	0.10
	
	GmaAffx.90824.1.S1_s_at	26S proteasome subunit RPN2a [HK]	mean	1.5	1.01	-1.06‡	-1.15	-1.03	-1.01	-1.06	-1.03	-1.07
			s.e.	0.20	0.11	0.04	0.09	0.06	0.13	0.09	0.06	0.04
	
	GmaAffx.90181.1.A1_at	Actin	mean	-1.36	1.49‡	-1.19‡	-1.29	-1.18	-1.28	-1.13	-1.33	1.10
			s.e.	0.26	0.40	0.06	0.10	0.22	0.16	0.12	0.13	0.22
	
	GmaAffx.60616.1.S1_at	Hcr9-OR2A	mean	-1.7	-2.2‡	-1.9‡	-1.45	-1.8	-2.0	-1.9	-1.7	-2.3
			s.e.	0.41	0.12	0.05	0.12	0.09	0.14	0.09	0.12	0.08
	
	Gma.447.1.S1_at	Defense associated acid phosphatase	mean	-1.43	-1.47	-1.8‡	-1.8	-1.6	-2.0	-1.8	-2.2	-1.48
			s.e.	0.14	0.21	0.06	0.15	0.12	0.17	0.13	0.09	0.17
	
	GmaAffx.64261.1.A1_at	Quinone oxidoreductase	mean	3.1	2.3‡	2.6‡	3.9	4.7	-1.24	2.7	3.0	3.0
			s.e.	0.6	0.40	0.39	1.5	1.5	0.23	0.8	1.1	1.1
	
	Gma.6640.1.S1_at	At5g66390 (secretory peroxidase)	mean	1.14	-1.08	-1.16‡	-1.38	-1.21	-1.20	1.30	-1.10	-1.33
			s.e.	0.49	0.22	0.06	0.15	0.10	0.15	0.16	0.17	0.13
	
	GmaAffx.18905.1.S1_at	Cell death associated protein	mean	3.4	1.5‡	2.9†‡	3.5	4.5	2.1	3.2	2.9	2.2
			s.e.	0.5	0.32	0.27	0.9	0.7	0.5	0.7	0.6	0.39
	
	Gma.17993.1.S1_at	P21 protein	mean	72	94‡	43a‡	41a	45a	44a	21b	100c	55a
			s.e.	27	27	7	18	20	16	9	24	17
	
	GmaAffx.75675.1.A1_at	Phenylalanine ammonia-lyase	mean	9.3	15‡	6.3‡	7.2	7.7	6.0	4.4	7.2	6.2
			s.e.	2.8	2.7	0.44	1.3	1.2	1.1	0.40	1.5	0.7
	
	GmaAffx.2799.1.A1_at	NtPRp27-like protein	mean	14	15‡	8.3‡	5.8	8.6	9.8	6.0	11	11
			s.e.	2.4	3.4	0.7	0.8	1.5	1.9	1.0	2.5	1.9
	
	Gma.16733.2.S1_at	WRKY4 transcription factor	mean	5.8	5.3‡	3.5‡	6.9	3.8	2.7	4.3	3.1	2.3
			s.e.	1.3	1.2	0.44	2.2	1.1	0.8	1.1	1.1	0.5
	
	GmaAffx.29692.1.S1_at	Chitinase (class II)	mean	2.5	2.7‡	3.1‡	3.2	2.7	2.7	2.9	3.5	4.2
			s.e.	0.35	0.6	0.21	0.6	0.34	0.6	0.27	0.7	0.5
	
	Gma.16911.1.S1_at	Cytochrome P450 family protein	mean	3.4	4.0‡	3.1‡	2.9	3.4	2.9	2.8	4.2	3.0
			s.e.	0.8	1.0	0.18	0.5	0.37	0.40	0.28	0.46	0.44
	
	GmaAffx.7209.1.A1_at	UDP-glucose 6-dehydrogenase	mean	1.8	2.2‡	1.02	1.49	1.28	-1.7	-1.08	1.16	1.04
			s.e.	0.27	0.6	0.11	0.21	0.28	0.12	0.14	0.31	0.26
	
	GmaAffx.47171.1.A1_at	Putative resistance protein	mean	-1.07	1.08	1.21‡	1.23	1.18	1.11	1.16	1.35	1.30
			s.e.	0.16	0.34	0.07	0.18	0.15	0.16	0.14	0.18	0.17
	
	Gma.3473.1.S1_at	Protein, small heat shock	mean	-1.41	-1.6	-2.2‡	1.03	-3.6	-3.3	-1.9	-2.4	-1.9
			s.e.	0.16	0.09	0.05	0.37	0.05	0.08	0.08	0.11	0.15
	
	GmaAffx.90984.1.A1_at	Germin-like protein	mean	5.3	2.5‡	-1.06	1.10	1.18	-1.18	-1.06	-1.12	-1.23
			s.e.	1.7	0.5	0.07	0.20	0.21	0.12	0.10	0.10	0.14
	
	GmaAffx.32612.1.S1_at	HI4'OMT	mean	57	16‡	25a‡	18a	22a	24a	18a	43b	41b
			s.e.	16	4.2	2.8	6	5	7	4.9	12	8
	
	GmaAffx.90059.1.S1_at	HcrVf2 protein	mean	3.9	2.5‡	2.7‡	5.5	4.0	1.3	2.3	2.8	2.4
			s.e.	1.1	0.38	0.25	0.8	0.7	0.17	0.35	0.44	0.7
	
	GmaAffx.79817.1.S1_at	SGT1	mean	1.21	-1.19	-1.04	1.08	-1.02	1.01	-1.02	-1.08	-1.18
			s.e.	0.28	0.05	0.05	0.18	0.06	0.20	0.07	0.05	0.04

^a^Genotypes used for this comparison were cultivars V71-370 and Sloan; ^b^22 genes selected for qRT assays. ^c^The functional annotation of the selected genes. [HK] indicates housekeeping genes used to normalize the qRT-PCR results ^d^The average expression difference between inoculated and mock samples, calculated as a fold-change. Negative values indicate downward change in response to infection. The presence of letter suffixes (a, b, c) after mean values indicates the presence of significant variation among the subgroup means, as determined by Least Significant Difference with a cutoff of an FDR-adjusted p value of 0.05. In those cases, means with the same suffix are not significantly different from one another. If no suffixes are present, there are no significant differences among any of the means. Symbol * indicates gene expression differences measured by microarray analysis in the pilot experiment that differ significantly from the measurements obtained by qRT-PCR analysis, determined using t tests with a cutoff of an FDR-adjusted p value of 0.05. Symbol † indicates gene expression differences measured by microarray analysis in the whole 72 replicate experiment that differ significantly from the measurements obtained by microarray analysis in the pilot experiment, determined using t tests with a cutoff of an FDR-adjusted p value of 0.05. Symbol ‡ indicates expression changes in the pilot microarray experiment and the whole 72 replicate experiment that are significant at a cutoff of a TST-FDR-adjusted p value of 0.01. ^e^The standard errors are calculated from log-transformed expression differences from 3 technical replicates in the case of the qRT-PCR results, from 4 biological replicates in the case of the pilot array data, from 72 biological replicates in the whole main experiment, and from 12 biological replicates in the main experiment sub-groups. ^f^All the 72-replicates as a whole experiment. ^g^Sub-groups of the whole experiment each comprising 12 consecutive replicates.

To further explore the biological reproducibility of the measured gene expression differences, we divided the 24 blocks into 6 sub-groups, each consisting of 4 consecutive blocks (12 measurements) and compared the results for each of the sub-groups for the same set of 22 genes (Table 5). Since the 24 blocks each constitute independent biological replicates spread over 8 months, each of the 6 sub-groups also constitute independent biological replicates. In most cases, only minor fluctuations were observed. Two genes showed significant fluctuations (Gma.17993.1.S1_at P21 protein and GmaAffx.32612.1.S1_at HI4'OMT; least significant difference, FDR adjusted p < 0.05).

### Comparing the power of large and small experiments

**Figure 1:**
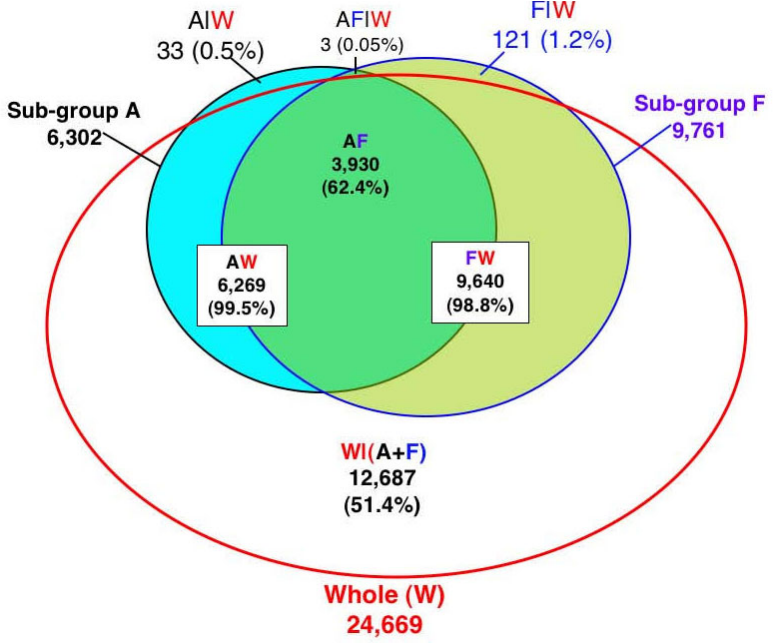
**Venn diagram showing the intersection between the sub-groups A and F and the whole experiment (W)**. Sub-group A is the first four blocks and F is the last four blocks of the whole experiment, which includes a total of 24 blocks. GC-RMA preprocessed data of A, F, and W were analyzed separately using the same LMMA model in SAS Proc Mixed. Genes with significant genotype × treatment interaction were determined using a cutoff of a TST-FDR adjusted p ≤ 0.01, as described in the methods. AW indicates the intersection between the A and W, FW the intersection between F and W, and AF the intersection of the two sub-groups A and F. W|(A+F) refers to genes in W but not in A or F, A|W to genes in A but not W, and F|W to genes in F but not W. AF|W refers to genes in A and F but not W; the three genes in this set were not found significant in the other four sub-groups (B, C, D, or E).

To compare the power of our experiment with experiments of a size comparable to those more commonly found in the literature, we again divided the 24 blocks into 6 sub-groups, each consisting of 4 consecutive blocks, and then reanalyzed the data by LMMA for each sub-group separately. As expected, each individual data set had less power than the overall data set. An average of 40.4%, 65.7% and 33.8% genes were detected as significant for genotype, treatment and genotype × treatment interaction, respectively, with TST-FDR control at a level of 0.01 [see Additional file 2]. However, different sub-experiments detected different subsets of the genes detected in the overall data set (Figure 1). Thus genes significant in all the 6 sub-groups comprised only 48.9% of those detected in the overall experiment [see Additional file 3]. This lack of agreement among the gene lists became worse at more stringent (lower) FDR levels. As is well-known, increased stringency produces more precise results (fewer false positives) but at the expense of a sometimes major decrease in power, i.e. an increase in the number of false negatives. However, the union of all genes that were significant in any of the six sub-groups comprised 81.0% of the genes with significant changes detected in the overall experiment [see Additional file 3]. These results demonstrate that the most common error made with small experiments is false negatives, rather than false positives, and the common practice of combining results of multiple experiments by only considering the intersection of the gene lists is unnecessarily extremely conservative. A simple union, the techniques of meta-analysis, or where possible a joint analysis, may be most appropriate for combining data from multiple independent experiments.

### Low-magnitude transcriptomic remodeling differs among functional categories

**Figure 2:**
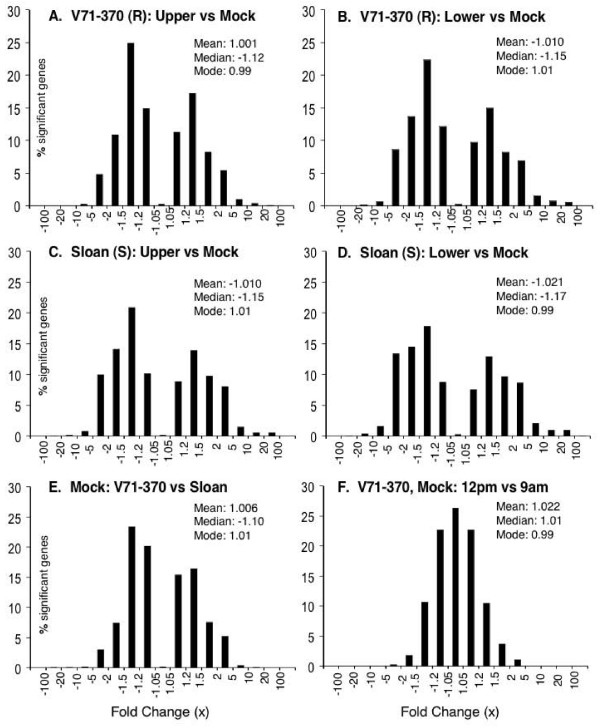
**Distribution of gene expression changes for different contrasts**. Only genes with significant changes (TST-FDR-adjusted p ≤ 0.01) are included. A negative fold-change indicates a reduction by that factor. *(A-D) *Infection responses in resistant genotype V71-370 and susceptible genotype Sloan. "Upper" and "lower" denote infection courts as described in the text and the methods. Distributions of gene expression changes for VPRIL9 infection responses are shown [see Additional file 4]. *(E) *Gene expression differences between V71-370 and Sloan following mock inoculation; genes with higher mRNA levels in V71-370 or Sloan are shown as having a positive or negative differences, respectively. For *(A)-(E)*, the fold changes were calculated using LMMA contrast analysis using GC-RMA normalized data. *(F) *Differences among 4,453 genes with significant responses to time of harvest in mock-inoculated Sloan plants: genes with higher mRNA levels in 12 noon samples or 9 am samples are shown as having a positive or negative differences, respectively. The fold changes were calculated from the average difference of the two treatments using the GC-RMA normalized data. The mean, median and mode of each distribution were calculated using the log-fold changes. The bin size of the mode was 0.01.

**Figure 3:**
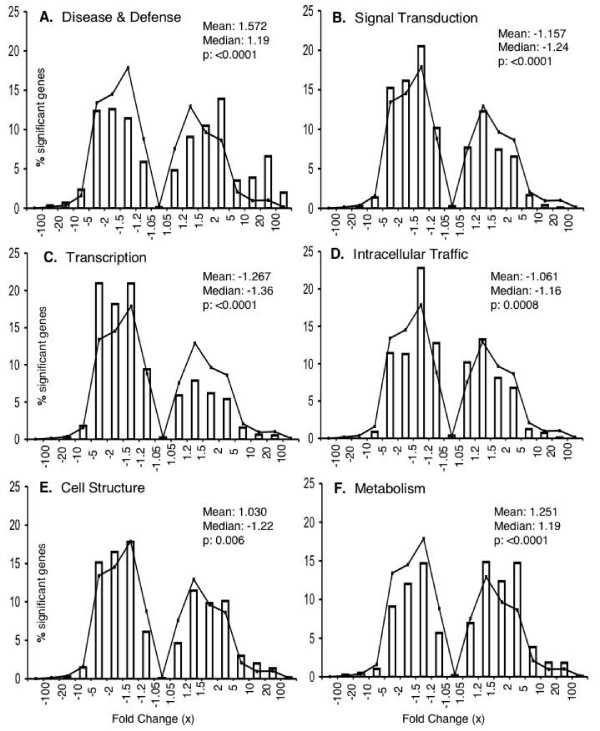
**Distributions of expression changes for genes within specific functional categories**. Six functional categories relevant to infection are shown. Distributions are shown for genes showing significant infection responses (Lower vs Mock) in Sloan as revealed by LMMA analysis of GC-RMA preprocessed microarray data with TST-FDR adjusted p value ≤ 0.01. Distribution comparisons for other cultivars and infection courts are shown [see Additional files 5 to 7]. Histograms show the number of genes in each fold change range. The line graph connects points corresponding to the numbers of genes in all categories in each fold change range. A negative fold change indicates a reduction in expression by that factor. p values in each panel indicate the result of a Kolmogorov-Smirnov test comparing the distribution of expression changes within the category to the distribution for all genes. The mean and median of each distribution were calculated using the log-fold changes.

Many researchers use two-fold change as an arbitrary cutoff. Our results show that 13.1 to 23.5% of the significantly changed genes in the upper infection court and 29.8% to 36.8% in the lower infection court changed by two-fold or greater in the three tested genotypes (Figure 2) [see Additional file 4]. However, 8.2 to 15.0% of the genes with significantly changes showed only subtle changes (fold change of less than 1.2-fold). To examine the functional significance of gene expression changes of various magnitudes, we plotted the distribution of changes for genes in six functional categories relevant to infection, namely defense and disease, signal transduction, transcription, intracellular trafficking, cell structure and metabolism (Figure 3) [see Additional files 5 to 7]. In the lower infection court of the susceptible cultivar, Sloan, which had the greatest level of infection, 23,556 genes showed significant changes in response to infection. Compared to the entire set of modulated genes, the distribution of changes was significantly different for all six categories (p ≤ 0.01, Kolmogorov-Smirnov test for two samples) (Figure 3). The disease and defense category and to a lesser extent metabolism showed a bias towards strongly up-regulated genes, whereas transcription and to a lesser extent signal transduction and intracellular trafficking, showed a bias towards down-regulation of genes. Similar patterns were observed in the upper infection court of Sloan and in both courts of the resistant cultivar V71-370 [see Additional files 5 to 8]. To test if significant changes in distribution were present among genes showing low magnitude modulation, the distribution comparison was restricted to genes showing less than two-fold or less than 1.5-fold modulation. Five of the categories (all except disease and defense), and three categories (transcription, cell structure and metabolism), respectively, showed significantly different distributions from the overall gene set (p ≤ 0.01). Even among genes with less than a 1.2-fold change, those in the transcription and metabolism categories still showed significantly different distributions (p ≤ 0.01) (Table 6) [see Additional file 8].

**Table 6:** Distributions of expression differences of genes in functional categories compared with the overall differences distribution

	Functional category^b^					
	
Fold change Range^a^	Disease & Defense	Signal Transduction	Transcription	Intracellular Traffic	Cell Structure	Metabolism
Full	< 0.0001^c^	< 0.0001	< 0.0001	0.0008	0.006	< 0.0001
± 2.0X	0.03	0.0002	< 0.0001	0.002	< 0.0001	< 0.0001
± 1.5X	0.34	0.018	< 0.0001	0.017	0.0003	< 0.0001
± 1.2X	0.57	0.37	0.0008	0.23	0.36	0.0004

^a^The fold changes were calculated by contrast analysis with SAS Proc Mixed using GC-RMA normalized data. "Full" = full range from the greatest down-regulation to the greatest up-regulation; "± 2.0X" = from down-regulated 2.0x to up-regulated 2.0x; "± 1.5X" = from 1.5x down-regulated to 1.5x up-regulated; "± 1.2X" = from -1.2x down-regulated to 1.2x up-regulated. ^b^Functional category of each gene drawn from annotation of the Affymetrix soybean GeneChip by the Goldberg group at the University of California, Los Angeles . ^c^p-value obtained by resampling for the Kolmogorov-Smirnov test for distribution differences between a particular functional category and the whole gene set for genes with significant infection responses in the lower court of Sloan; p values were adjusted for FDR by the TST method. Results for other cultivars and infection courts are shown [see Additional file 8].

## Discussion

Infection profoundly affects the physiology of host cells, including the levels of mRNAs within each cell. The ability to measure changes in host mRNA levels during infection is greatly limited by the intrinsic variability of the tissue and the difficulty of accurately reproducing infection across multiple replicates. As result, typical studies report significant changes in only a few percentage of the genes being assayed [3-5,7,8,10]. Similar difficulties limit the power of many other experiments that measure transcriptional responses to genetic and physiological perturbations. Here we have shown, by means of an experiment of unprecedented statistical power, that significant changes in the levels of nearly all host transcripts can be measured during infection of soybean with the eukaryotic pathogen *Phytophthora sojae*. Up to 36.8% of the changes were greater than 2-fold in magnitude but the majority were of lower magnitude. Up to 15% of the changes were less than 1.2-fold in magnitude. Similar widespread reprogramming of the transcriptome was also detected in response to genotypic differences among soybean cultivars, and even in response to different times of the day at which plants were harvested.

An experiment such as this, combining 72 biological replicates, each comprised of 20–30 plants, is very successful at identifying overall trends, in this case identifying overall responses to pathogen infection. In many of the individual genes we examined, the overall measurement also was a very good representation of a consistent response to infection observed across many independent experiments. On the other hand, some genes showed significant fluctuations from experiment to experiment. A major challenge in the future will be to design experiments that can dissect these changes and ask whether the fluctuations simply result from technical variations in the assay, whether the fluctuations result from small uncontrolled variations in the conditions in which the biological material is grown, or the more interesting possibility that fluctuations in mRNA are a normal physiological event. It is possible for example that organisms have evolved the ability to tolerate significant changes in the levels of mRNAs or other cellular components, and there is no evolutionary advantage to imposing extremely ranges on mRNA levels.

By re-analyzing subsets of our data that represented the scale of replication more commonly found in microarray experiments (4 replicates), we showed that approximately half of the transcriptional changes that we detected could not be observed with the small-scale experiments. Taking the intersection of six independent subsets greatly reduced the number of detected changes further, showing that this common practice for combining the results of multiple experiments is extremely and unnecessarily conservative. On the other hand, taking the union of multiple gene lists more closely approximated the results from the full-scale experiment.

A recent expression QTL study in *Saccharomyces cerevisiae *showed variations in the levels of 79%, 69% and 47% of the detectable transcripts in response to treatment, genotype and treatment × genotype interactions respectively [15]. Those findings, together with ours, suggest that widespread, low-magnitude transcriptional remodeling may be a normal process during physiological adaptation in eukaryotes, but one that is missed by conventional experimental designs. The extensive treatment × genotype interactions observed in both these studies suggest that the transcriptional reprogramming is genetically controlled.

The overall physiological response of an organism or cell to a stimulus may require coordinated changes in a wide array of cellular components. Those changes in turn may require compensating or reinforcing changes in an even wider array of functionally-connected cellular components. As a result, understanding how a specific set of transcriptional changes relates causally to a physiological change at a whole system level, is a major challenge. Our analysis suggests that low magnitude expression changes may be of functional significance. The observation that the patterns of low-magnitude transcriptional remodeling were significantly different among most functional categories, in some cases even among genes with less than 20% perturbation, is consistent with the hypothesis that low amplitude remodeling has functional significance. However, we cannot rule out that some percentage of genes may show low magnitude transcriptional modulation that has no functional significance, that is, they represent a low uncontrollable level of transcriptional variation that soybean (and other organisms) may have evolved to tolerate. Interestingly, most of the transcriptional remodeling of defense and disease response genes was of high magnitude, while low-magnitude remodeling was widespread in the other functional categories. We speculate that strong transcriptional modulation of disease and defense-related genes is required for the host to directly engage the pathogen, while the numerous other genes that function in other categories are coordinately modulated to support or adjust to the direct response.

Biological gene regulatory networks are highly interconnected systems. Non-linear, synergistic interactions are common. Large numbers of genes with low magnitude transcriptional modulation could potentially be just as important in conferring phenotypes and mediating physiological adaptation as the small numbers of genes that show large magnitude modulations. However, understanding the role of pervasive low magnitude remodeling may require using computational modeling approaches at a systems level, as well as improved technologies for accurately and cheaply measuring those changes. Systems approaches will also be needed to develop a deeper understanding of how consistent small magnitude changes and stochastic fluctuations are integrated to produce phenotypes.

## Conclusion

Our findings, consistent through two different normalization methods and two different statistical analysis procedures, suggest that almost the entire soybean genome undergoes significant transcriptional remodeling in response to pathogen infection and genetic variation. The majority of the transcriptional differences are less than two-fold in magnitude. The low amplitude modulation of gene expression (less than two-fold changes) is highly statistically significant, even for modulations of less than 20%. These findings demonstrate that low amplitude transcriptional modulation forms an integral component of physiological adaptation in soybean, and, we speculate, in all eukaryotes.

## Methods

### Plant materials and pathogen isolate

Three soybean genotypes with varying degrees of resistance to *Phytophthora sojae *were used for this study. V71-370 is a resistant genotype, VPRIL9 is moderately susceptible and Sloan and is highly susceptible[16,17]. *P. sojae *isolate, PT2004C2.S1, isolated from Ohio in 2004, and virulent to soybean differentials with *Rps1a*, *Rps1b*, *Rps1k*, *Rps2*, *Rps3a*, *Rps3c*, *Rps4*, *Rps5*, *Rps6*, or *Rps7*, was used in this study.

### Inoculation assay

Inoculation assays were performed using the slant board technique [16]. Briefly, 7-day-old soybean seedlings, grown in vermiculite, were thoroughly rinsed under tap water and placed in inoculation trays (20–30 plants per sample). The plants were wounded at 2 cm below the beginning of the root zone by scraping the epidermis with a scalpel and then were inoculated with a mycelial agar slurry from a 7-day-old culture or agar alone. Root tissue samples 7.5 mm long were collected at 5 days post inoculation (dpi) from immediately below (the treatment "Lower") and above (the treatment "Upper") the upper lesion margin from each seedling with characteristic lesions. In each case, the root tissue samples from like treatments and harvests were pooled. Thus each set of 30 pathogen-inoculated plants yielded two pooled samples, one pooled from 30 sections from the lower infection courts and one pooled from 30 sections from the upper infection courts. For the mock-inoculated plants (the treatment "Mock"), 15 mm tissue sections were taken spanning from 7.5 mm below to 7.5 mm above the position corresponding to the average lesion length measured from the inoculated samples. Thus each set of 20 mock-inoculated plants yielded one pooled mock sample. All the plants for each block were grown in the same growth chamber (Environmental Growth Chambers, Chagrin Falls, Ohio; Model M-48 with TC2 microcontroller unit) with day and night temperatures settings of 27°C and 21°C, respectively and relative humidity averaging 75 to 90%.

### Overall experimental design

The data for this study comes from control inoculations that formed part of a large study of a recombinant inbred line population that was organized into 29 blocks. All plants for a block were raised, inoculated and incubated together in the same growth chamber. Each block included the same three control genotypes (V71-370, Sloan and VPRIL9). Three replicates of the three genotypes were inoculated with *P. sojae *(generally 30 plants) or mock-inoculated (generally 20 plants), then harvested 5 days later at three different times during the morning of harvest (9 am, 10:30 am, and 12 pm). Each block yielded 27 sets of samples, which were the complete combinations of the 3 genotypes, 3 treatments and 3 time windows. Thus the experiment was balanced with respect to the factors, soybean genotypes, treatment ("Upper", "Lower", and "Mock") and inoculation time window. Since the differences between the samples harvested at 9 am, 10:30 am and 12 pm were small, in this study we refer to the 3 × 29 sets of samples obtained from the 29 blocks as biological replicates. The 24 blocks in which all the control samples passed quality control were used for statistical analyses.

### RNA extraction and microarray assay

Total RNA was isolated from each of the pooled plant samples using the QIAGEN RNeasy^® ^Plant Mini Kit, following the manufacturer's protocol for total RNA extraction from plant and filamentous fungal tissue with the following minor modifications. 250 mg of fine tissue powder was used with appropriate scaled-up buffer volumes, and the Buffer RLT-suspended samples were thoroughly vortexed for 2 minutes and then incubated in 56°C water-bath for 3 min and kept in ice for 1 min before the first spin. The quantity and quality of total RNA were checked with both a NanoDrop ND-1000 Spectrophotometer and an Agilent 2100 Bioanalyzer.

Microarray procedures were performed at the Core Laboratory Facility (CLF) of the Virginia Bioinformatics Institute. The standard eukaryotic gene expression assay protocols were followed as described in the Affymetrix GeneChip^® ^Expression Analysis Technical Manual. Briefly, as the first step, biotin-labeled cRNA was generated using the One-Cycle Target Labeling and Control Reagents (Affymetrix) and 1 μg of total RNA. Second, 20 μg of biotin-labeled cRNA was fragmented in Fragmentation Buffer. Third, fragmented cRNA was hybridized to a soybean GeneChip at 45°C for 16 h in an Affymetrix hybridization oven (model 640). Fourth, the GeneChips were washed and stained with streptavidin-phycoerythrin using the fluidics protocol EukGE-WS2v5-450 in the Affymetrix^® ^GeneChip^® ^Fluidics Station 450. Finally, the stained chips were scanned with an Affymetrix GCS3000 7G Scanner. The Affymetrix GeneChip^® ^Operating Software (GCOS, v1.4.0.036) was used to provide instrument control, first-level data analysis, and data management for the entire GeneChip System. Samples within experimental blocks were randomized before being provided to the CLF, to reduce possible block effects arising from processing samples together during the microarray analysis work flow.

### Low level data pre-processing

Quality control was performed using a variety of tools from the MAS5 and AffyPLM quality control toolsets. These included analysis of percentage of probes called present, the scaling factor and average background of each chip. In addition, ratios of 3' to 5' probes for β-Actin were used to check for RNA degradation. Calculation of the interquantile ranges of the normalized unscaled standard error and of the relative log expression was used to remove outliers. All chips in the 24 blocks selected for this study passed these QC tests. Low-level analysis of the raw GeneChip data began with filtering the non-expressed genes based on the Affymetrix Microarray Suite version 5 (MAS5) algorithm for calls (as implemented in the Bioconductor package *affy*, ) taking into account the experimental design. The default parameter τ (0.015) in the MAS5 present call was used, and a probe set (which approximately represents a gene, as 95% of the soybean probe sets are unique) was declared to be detectable (or present) if it had a present call in 40% of the chips.

The principal data pre-processing method used in our data analyses was GC-RMA, which included a GC-RMA background correction, quantile normalization, and computation of gene summary values from the corrected probe-level data. Background correction was performed with the model-based procedure [18] using sequence information as implemented in the *Bioconductor *package *gcrma *. Quantile normalization [19] and Tukey's median-polish algorithm based gene summary values were computed using a modified C program capable of processing several thousand GeneChips simultaneously (L. Bao, unpublished results).

As an alternative method for data pre-processing, we used the Affymetrix MAS5 default algorithms available in the *affy *package. Global scaling was implemented to assure that all the chips had the same trimmed mean signal intensity using all soybean probe sets.

Our finding that most detectable transcripts show significant changes raises important questions about the assumptions underlying normalization procedures. MAS5 assumes that the sum of the expression levels of all transcripts remains constant. Quantile normalization assumes this also, and assumes in addition that the distribution of probe signals remains constant across all arrays. Invariant set approaches assume that a pre-determined set of genes show no changes. The assumption that the sum of the expression levels of all transcripts remains constant is a highly robust one because the amount of labeled cRNA added to a microarray hybridization is always normalized. Thus even if the amount of mRNA per cell in the source tissue varied (which should be reflected in variation in the sum of the expression levels of all transcripts) this variation is cancelled by the experimental procedure. Accurately capturing changes in the amount of mRNA per cell in source tissue is currently technically infeasible.

To estimate whether normalization had systematically biased the measured transcriptional changes, we determined the mode of the changes. Under the assumption that the most common transcriptional changes will be the smallest in magnitude, the mode should lie close to 1.0 (i.e. no change). In fact the mode in each contrast studied ranged between 0.99 and 1.01.

### Linear mixed model analysis of gene expression levels

A total of 648 soybean GeneChips from 24 complete experimental blocks were selected for the high-level data analyses. The high-level analyses were performed separately for each soybean probe set, using Linear Mixed Model Analysis (LMMA) in the PROC MIXED procedure of SAS (SAS 9.1 for Windows, SAS Institute Inc., Cary, NC, USA) [20]. The fixed factors of the linear mixed model included genotype, treatment (Upper, Lower and Mock), and time (9 am, 10:30 am, and 12 pm), and the genotype by treatment interaction. Random factors included in the model were: block, the interactions block by genotype, block by treatment, block by time and block by genotype by treatment, and residual. Variance components were estimated by the method of Residual Maximum Likelihood, and F-tests were performed for all fixed factors.

All F-tests across all genes and all the fixed factors were considered as one family of tests, and the resultant collection of p-values was used to compute adjusted p-values based on two False Discovery Rate (FDR) controlling methods, the linear step-up method of Benjamini and Hochberg [21] and the two-stage linear step-up method (TST) of Benjamini et al [22]. In addition, the p-values were used to compute the q-values for the positive FDR criterion as described by Storey and Tibshirani [23]. Because the differences between the FDR methods were small, we mainly report results obtained with the TST method.

We also estimated and tested individual contrasts of interest, most of which were linear functions of the cultivar by treatment interactions. Because the F-test for the genotype by treatment interaction was found to be significant for the majority of probe sets (24,669 out of 28,351), we employed a one-step approach for testing the significance of individual contrasts, i.e. we tested all contrasts of interest for all genes rather than for only those genes with significant F-test for the genotype by treatment interaction. We were interested in identifying the differentially expressed genes for the contrast "infection response", which compares gene expression between pathogen and mock inoculation in a given cultivar and infection court, and these are used to determine which genes are up- or down-regulated within a given infection court as a result of the pathogen attack in a given cultivar. The infection responses were split into two subtypes, i.e., Upper vs. Mock, and Lower vs. Mock. The collection of the t-tests for all contrasts across all genes was considered as a family of tests, and significant contrasts were determined with the same FDR methods as those used for the F-tests.

### Wilcoxon signed rank tests of differential gene expressions

As an alternative high-level analysis to LMMA, we used the non-parametric Wilcoxon Signed Rank Test using the wilcox.test function (R package *stats *version 2.6.0). For each gene and within each cultivar, we formed two pairs of samples, Upper and Mock, and Lower and Mock, respectively. Each consisted of the GC-RMA log2 scale signal intensity data for the paired treatments (e.g. Upper and Mock) (sample size = 3 × 24 = 72). The collection of p-values across all genes was considered as a family of tests, and significant contrasts were determined with the same FDR methods as those used earlier. The same procedure was used to perform the Wilcoxon signed rank test and FDR control with the preprocessed data generated by MAS5.

### Kolmogorov-Smirnov test for two distribution patterns

We used the Kolmogorov-Smirnov (KS) test to test for significant differences in the distributions of gene expression changes for specific categories of genes. The expression changes in response to pathogen inoculation were considered for genes in which the change was significant by LMMA contrast analysis of infection responses using SAS Proc Mixed. The distributions of expression changes were examined for genes in six functional categories related to infection: "defense and disease", "signal transduction", "transcription", "intracellular trafficking", "cell structure" and "metabolism". For this purpose we used the functional category of each gene drawn from annotation of the Affymetrix soybean GeneChip by the Goldberg group at the University of California, Los Angeles ; we have reviewed this annotation extensively and found it very reliable. In each case, the distribution within the functional category was compared to the distribution for the total gene set, including the genes within the functional category, to test the null hypothesis that the observed distribution of expression changes affected all genes irrespective of functional category. This is a more conservative test than testing the distribution of a subset of genes against the remainder of the genes. To test for distribution differences within specific ranges of gene expression changes, three ranges were chosen: all genes with less than two-fold regulation (up or down); all genes with less than 1.5-fold regulation (up or down); and all genes with less than 1.2-fold regulation (up or down). Then the distribution of changes was compared between a functional category and all genes within the chosen expression range. For all these tests we used the ks.test function in the R package *stats *(version 2.7.0). Because we compared the distribution of the genes in a category to that of all genes, and because the gene expression profiles have complex correlation structures and thus the values for individual genes may not be independent [24], we computed an empirical p value for each test by the following procedure. For any gene category with n genes, we randomly sampled n genes from the list of all significant genes and recomputed the KS test statistic. This process was repeated 10,000 times, and the collection of ordered KS statistics was then used to compute an empirical p-value for the category. Finally, FDR control was performed on the set of p-values from all KS tests using the TST method [22].

### Quantitative real time RT-PCR (qRT-PCR) assay

To provide a comparison between the microarray assays and another commonly used measure of gene expression differences, qRT-PCR, gene expression differences (infected versus mock) for a set of selected genes were measured by qRT-PCR during a pilot experiment. The pilot experiment included four biological replicates and was conducted using the same methods as described above, with the following modifications: for each of the two cultivars (V71-370 and Sloan) four replicates of 30 plants (grown together in the same growth chamber) were harvested 5 days after inoculation, and a single segment of infected tissue (treatment "Inoculated") comprising the upper and lower infection courts was excised from each plant. RNA was extracted from each pool of 30, then equal amounts of the RNAs were pooled from four biological replicates for microarray analysis. In parallel, an equal number of mock-inoculated plants were harvested and RNA extracted pooled in the same way.

For qRT-PCR assays, 22 soybean genes of interest (including 4 housekeeping (HK) genes) with varied levels of gene expression were selected (Table 5). Equal amounts of RNA from each of the four biological replicates were pooled for the qRT-PCR assays. The primers [see Additional file 9] were designed using the Beacon Designer 4.0 (Premier Biosoft International, Palo Alto, CA) and synthesized by Integrated DNA Technologies, Inc.(Coralville, IA). The amplicons for development of standard curves were prepared first using the purified total RNA as a template and the SuperScript™ III First-Strand Synthesis System for RT-PCR (Invitrogen™ Life Technologies) and then using conventional PCR with the synthesized cDNA as template. The amplicons were purified using a QIAquick PCR Purification Kit (Qiagen) and checked for quality in the Bioanalyser.

qRT-PCR assays were carried out by the Virginia Bioinformatics Institute Core Laboratory Facility. Briefly, total RNA (1 μg) was transcribed to cDNA using first strand cDNA synthesis reagents (Invitrogen Corp., Carlsbad, CA) in a total volume of 20 μl. Standard curves were produced with serial 10-fold dilutions of cDNA products starting from 10 pg/μl. Each 25 μl qRT-PCR reaction consisted of 300 nM sense and anti-sense primers, 1 μl 10× diluted cDNA, and 12.5 μl SYBR Green I PCR Mastermix (Applied Biosystems, Foster City, CA). Each reaction was run in triplicate for both the standard and samples. PCR reactions were performed on a Bio-Rad i-cycler (BioRad, Hercules, CA) under the following conditions: 95°C for 3 min, 40 cycles of 95°C for 10 s, 56°C for 45 s to calculate cycle threshold (CT) values, followed by 95°C for 1 min, 55°C for 1 min, and 80 times of 55°C for 10 s, increasing temperature by 0.5°C each cycle to obtain melt curves. The BioRad iCycler IQ 3.1 Optical System Software was used and PCR efficiency (E) was estimated using the equation (1+E) = 10^(-1/slope) ^[25]. Pathogen-inoculated vs Mock expression ratio of a gene of interest was calculated from the equation:

$$\text{Ratio}=\frac{{\left(1+E_{gene}\right)}^{\Delta CTgene}}{NF}$$

Where *E *denotes PCR efficiency, *CT *denotes cycle threshold value, *ΔCT *is the cycle threshold difference between a mock sample and its corresponding pathogen-inoculated sample, *gene *denotes the gene of interest, *NF *denotes the normalization factor calculated from the geometric mean of the raw ratios (i.e., (1 + *E*_*h*_) ^Δ*CTh*^, here *h *denotes a house keeping gene) of the three selected housekeeping genes [26]. Four housekeeping genes (the first four rows of Table 5, including actin) were initially evaluated, based on the microarray data from the pilot experiment. The best three (marked [HK] in Table 5) were selected using the gene-stability measure and ranking method [26].

## Availability

The microarray data discussed in this publication have been deposited in NCBI's Gene Expression Omnibus [27] and are accessible through GEO Series accession number GSE11611 .

## Authors' contributions

Writing team: BMT, LZ, IH, AED; Project management: BMT, MASM., IH., AED, SKSM, LZ, ST; Inoculation assays and sampling: SXM, AED; RNA preparations and microarray assays: LZ, RH, FDA, KK, CE, AJ; Database management: ST, LZ, KK; Statistical analyses: LZ, LB, BMT, IH. All authors have read and approved the final manuscript.

## Supplementary Material

Additional file 1 **Supplemental Figure 1.** Correlation among gene expression changes in response to *P. sojae *infection measured in independent experiments by qRT-PCR and microarrays. The data provided compare gene expression changes in response to pathogen infection measured in two experiments 2 years apart, and the correlation between qRT-PCR and microarray measurements. Click here for file

Additional file 2 **Supplemental Table 1.** Intersection of six non-random sub-groups with the whole experiment. The data describe the intersections among six independent sub-groups of the whole experiment at different FDR (TST) levels. Click here for file

Additional file 3 **Supplemental Table 2.** Comparisons among the intersection or union of the sub-groups and the whole experiment. The data demonstrate the differences in the power between smaller experiments and the complete experiment, and different ways of combining the results of the smaller experiments. Click here for file

Additional file 4 **Supplemental Figure 2.** Distribution of gene expression changes during infection of genotype VPRIL9. The data describe the fractions of genes showing different levels of gene expression changes during infection of moderately susceptible genotype VPRIL9. Click here for file

Additional file 5 **Supplemental Figure 3.** Distributions of expression changes in the Sloan upper infection court among six functional categories. The data describe the fractions of genes with different expression changes in the Sloan upper infection court within specific functional categories. Click here for file

Additional file 6 **Supplemental Figure 4.** Distributions of expression changes in the V71-370 lower court among six functional categories. The data describe the fractions of genes with different expression changes in the V71-370 lower court within specific functional categories. Click here for file

Additional file 7 **Supplemental Figure 5.** Distributions of expression changes in the V71-370 upper court among six functional categories. The data describe the fractions of genes with different expression changes in the V71-370 upper court within specific functional categories. Click here for file

Additional file 8 **Supplemental Table 3.** Distributions of expression differences of genes in functional categories compared with the overall differences distribution. Detailed data summarizing numbers of genes with different expression differences in six functional categories compared with the overall distribution, in two cultivars and both infection courts. Click here for file

Additional file 9 **Supplemental Table 4**. Genes and primers used for quantitative real time RT-PCR assay. The Affymetrix ID, functional annotation and primer sequences of the 22 genes used in the qRT-PCR assay. Click here for file
