# Supplementary material for: Infection and genotype remodel the entire soybean transcriptome
Source: BMC Genomics. 2009 Jan 26;10:49. doi: 10.1186/1471-2164-10-49 (PMC2662884; doi:10.1186/1471-2164-10-49)
Supplement: Additional file 1 — Supplemental Figure 1. Correlation among gene expression changes in response to P. sojae infection measured in independent experiments by qRT-PCR and microarrays. The data provided compare gene expression changes in response to pathogen infection measured in two experiments 2 years apart, and the correlation between qRT-PCR and microarray measurements. [file 1471-2164-10-49-S1.pdf]

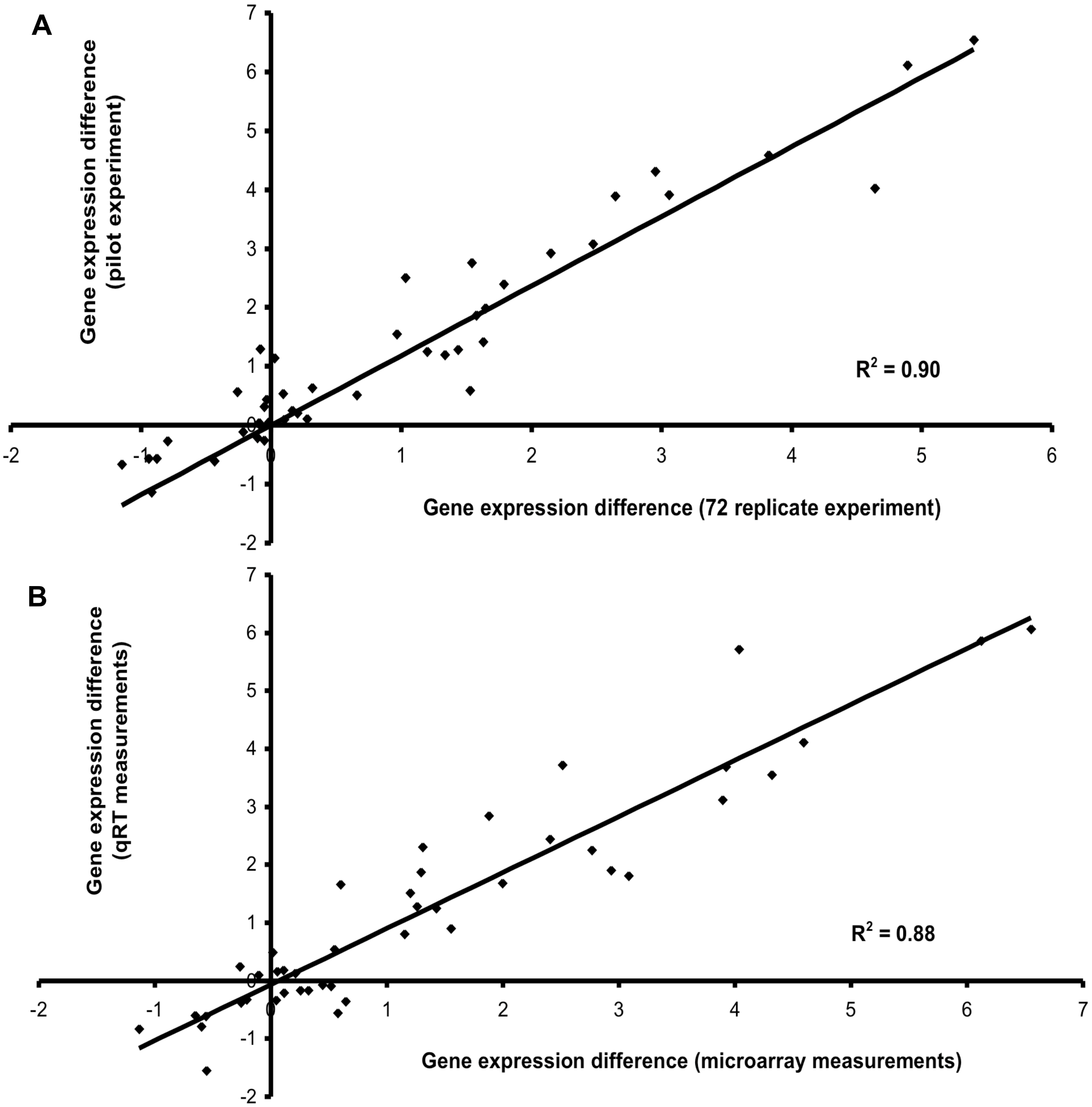

Supplemental Figure 1. Correlation among gene expression changes in response to *P. sojae* infection measured in independent experiments by qRT-PCR and microarrays. (A) Gene expression differences (infected versus mock) for 22 selected genes, compared between the 72 replicate experiment and a pilot experiment conducted two years' previously. (B) Gene expression differences (infected versus mock) for the 22 selected genes; comparison of microarray and qRT-PCR measurements using RNA from the pilot experiment. The pilot experiment was repeated four times. Correlation in gene expression differences between the two experiments was 0.85 (R^2) for all detectable genes and 0.90 (R^2) for the selected genes. For qRT-PCR comparison, 22 soybean genes of interest (including 4 housekeeping (HK) genes) with varied levels of gene expression were selected. The genes annotated as Ubiquitin (AtRUB1) (Gma.441.1.S1_at), ribosomal protein S27-like protein (Gma.16125.1.S1_s_at), and 26S proteasome subunit RPN2a (GmaAffx.90824.1.S1_s_at) were ranked as top three house keeping genes using the gene-stability measure and ranking method. The geometric mean of the top three housekeeping genes were used to normalize the qRT-PCR measurements of all the 22 selected genes. For qRT-PCR analysis, equal amounts of RNA samples from the four replicates were pooled. qRT-PCR assays were carried out by the Virginia Bioinformatics Institute Core Laboratory Facility.
