# Supplementary material for: Infection and genotype remodel the entire soybean transcriptome
Source: BMC Genomics. 2009 Jan 26;10:49. doi: 10.1186/1471-2164-10-49 (PMC2662884; doi:10.1186/1471-2164-10-49)
Supplement: Additional file 2 — Supplemental Table 1. Intersection of six non-random sub-groups with the whole experiment. The data describe the intersections among six independent sub-groups of the whole experiment at different FDR (TST) levels. [file 1471-2164-10-49-S2.pdf]

Supplemental Table 1. Intersection of six non-random sub-groups with the whole experiment.

Experiment[a]		Genotype x Treatment[b]			Genotype[b]			Treatment[b]		
		0.05[c]	0.01	0.001	0.05	0.01	0.001	0.05	0.01	0.001
Whole (W)	Significant [d]	26,500	24,669	22,381	27,156	25,923	24,191	27,732	27,139	26,351
Sub A	Significant	11,710	6,302	2,769	14,930	7,961	3,583	20,902	16,296	11,138
	Intersection[e]	11,635	6,269	2,761	14,842	7,913	3,543	20,855	16,277	11,118
	% of A[f]	99.4	99.5	99.7	99.4	99.4	98.9	99.8	99.9	99.8
	% of W[f]	43.9	25.4	12.3	54.7	30.5	14.6	75.2	60.0	42.2
Sub B	Significant	13,292	8,225	4,366	15,263	8,655	3,852	21,879	17,374	11,835
	Intersection	13,252	8,210	4,363	15,224	8,640	3,845	21,870	17,369	11,830
	% of B	99.7	99.8	99.9	99.7	99.8	99.8	100	100	100
	% of W	50.0	33.3	19.5	56.1	33.3	15.9	78.9	64	44.9
Sub C	Significant	15,913	10,610	6,114	17,421	10,891	5,307	23,124	19,452	14,854
	Intersection	15,755	10,516	6,081	17,378	10,863	5,287	23,088	19,419	14,836
	% of C	99.0	99.1	99.5	99.8	99.7	99.6	99.8	99.8	99.9
	% of W	59.5	42.6	27.2	64.0	41.9	21.9	83.3	71.6	56.3
Sub D	Significant	15,834	10,917	6,348	19,559	13,968	7,508	23,470	19,879	14,377
	Intersection	15,731	10,850	6,331	19,519	13,935	7,480	23,440	19,867	14,369
	% of D	99.3	99.4	99.7	99.8	99.8	99.6	99.9	99.9	99.9
	% of W	59.4	44.0	28.3	71.9	53.8	30.9	84.5	73.2	54.5
Sub E	Significant	17,081	11,666	6,611	20,461	13,554	6,508	22,812	18,590	12,739
	Intersection	16,961	11,603	6,590	20,270	13,405	6,417	22,776	18,568	12,733
	% of E	99.3	99.5	99.7	99.1	98.9	98.6	99.8	99.9	100
	% of W	64.0	47.0	29.4	74.6	51.7	26.5	82.1	68.4	48.3
Sub F	Significant	15,270	9,761	5,402	19,952	13,767	7,774	23,697	20,229	15,568
	Intersection	15,064	9,640	5,344	19,846	13,704	7,739	23,650	20,190	15,546
	% of F	98.7	98.8	98.9	99.5	99.5	99.5	99.8	99.8	99.9
	% of W	56.8	39.1	23.9	73.1	52.9	32.0	85.3	74.4	59.0

[a]The whole experiment (W) was split into six sub-groups (Sub A, B, C, D, E, and F, each with 4 blocks), in the order the blocks were performed. [b]Genotype x Treatment, Genotype (V71-370, Sloan and VPRIL9) and Treatment (Upper, Lower, and Mock) were fixed factors used in the linear mixed model analysis. Model: y = Genotype + Treatment + Time + Genotype x Treatment + Block + Block x Genotype + Block x Treatment + Block x Time + Block x Genotype x Treatment + Error, where y refers to the log2 scale median polish summarized gene expression values, the main factors (Genotype,

Treatment and Time) and the interaction Genotype x Treatment were fixed factors while all the remaining terms were random factors. [c]FDR (TST) adjusted p value cutoffs used to determine significance. [d]The number of genes significant for the factor of interest at the indicated cutoff from the whole experiment or a sub-group experiment, as revealed by LMMA analysis of GC-RMA preprocessed data. [e]Intersection indicates the number of genes significant in both the whole experiment (W) and in the relevant sub-group experiment. [f]Number of significant genes in the intersection as a percentage of the number of significant genes from the sub-group (e.g. A) or the whole experiment (W), respectively.
