# Supplementary material for: Infection and genotype remodel the entire soybean transcriptome
Source: BMC Genomics. 2009 Jan 26;10:49. doi: 10.1186/1471-2164-10-49 (PMC2662884; doi:10.1186/1471-2164-10-49)
Supplement: Additional file 3 — Supplemental Table 2. Comparisons among the intersection or union of the sub-groups and the whole experiment. The data demonstrate the differences in the power between smaller experiments and the complete experiment, and different ways of combining the results of the smaller experiments. [file 1471-2164-10-49-S3.pdf]

Supplemental Table 2. Comparisons among the intersection or union of the sub-groups and the whole experiment

FDR[a]	Fixed Factor[b]	Whole[c]	Intersection[e]		Union[g]			
		Significant[d]	significant	Matches[f]	significant	Matches[h]	% of union[i]	% of whole[j]
0.05	Genotype	27,156	8,845	8,845	25,969	25,543	98.4	94.1
	Treatment	27,732	16,914	16,914	27,012	26,845	99.4	96.8
	G x T	26,500	5,424	5,424	24,994	24,383	97.6	92
	Time	9,960	43	43	8,313	5,034	60.6	50.5
0.01	Genotype	25,923	3,107	3,107	21,633	21,326	98.6	82.3
	Treatment	27,139	11,137	11,137	24,944	24,829	99.5	91.5
	G x T	24,669	2,321	2,321	19,323	18,952	98.1	76.8
	Time	4,453	9	9	2,594	1,457	56.2	32.7
0.001	Genotype	24,191	460	460	16,148	15,937	98.7	65.9
	Treatment	26,351	4,174	4,174	22,388	22,312	99.7	84.7
	G x T	22,381	597	597	12,922	12,785	98.9	57.1
	Time	2,025	0	0	806	480	59.6	23.7
0.0001	Genotype	22,611	84	84	12,233	12,090	98.8	53.5
	Treatment	25,560	839	839	20,141	20,084	99.7	78.6
	G x T	20,293	138	138	8,803	8,737	99.3	43.1
	Time	1,173	0	0	372	226	60.8	19.3

[a]FDR refers to p value cutoff following false discovery rate control with the two-stage linear step-up method (TST-FDR). [b]The fixed factors used in the LMMA model included genotype (V71-370, Sloan and VPRIL9), treatment (Upper, Lower, and Mock), time (9 am, 10:30 am, and 12 pm), and the genotype x treatment interaction (G x T)). [c]The whole 24 block experiment. [d]The number of significant genes revealed by LMMA analysis of GC-RMA preprocessed data at the designed p value cutoff. [e]The number of significant genes found in all the 6 sub-groups. [f]The number of genes found significant in both the intersection of all the sub-groups and in the whole experiment. No genes were found in the intersection but not the whole. [g]The number of genes found significant in at least one of the sub-group experiments. [h]The number of genes found significant in both the Union of the sub-group results and in the whole experiment. [i]The number of matching genes as a percentage of the total number of significant genes in the Union. [j]The number of matching genes as a percentage of the total number of significant genes discovered in the whole experiment.
