# Supplementary material for: Infection and genotype remodel the entire soybean transcriptome
Source: BMC Genomics. 2009 Jan 26;10:49. doi: 10.1186/1471-2164-10-49 (PMC2662884; doi:10.1186/1471-2164-10-49)
Supplement: Additional file 4 — Supplemental Figure 2. Distribution of gene expression changes during infection of genotype VPRIL9. The data describe the fractions of genes showing different levels of gene expression changes during infection of moderately susceptible genotype VPRIL9. [file 1471-2164-10-49-S4.pdf]

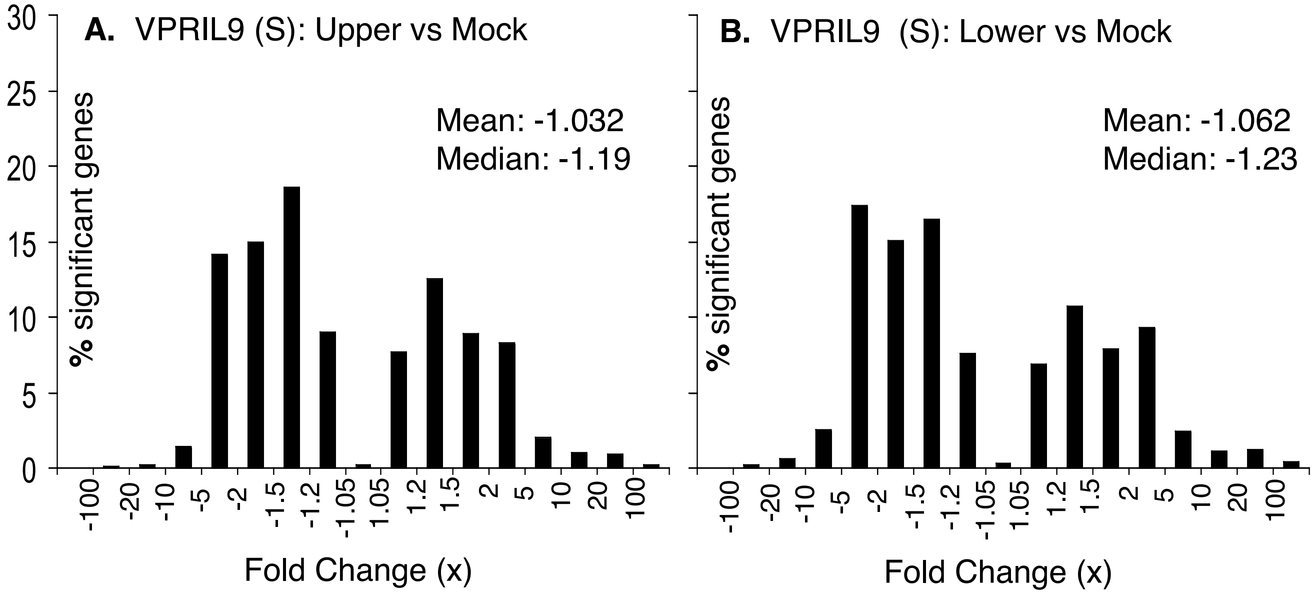

Supplemental Figure 2. Distribution of gene expression changes during infection of genotype VPRIL9. *(A)* upper infection court; *(B)* lower infection court. Only genes with significant changes (TST-FDR-adjusted $p \leq 0.01$) are included. A negative fold-change indicates a reduction by that factor. The fold changes were calculated using LMMA contrast analysis with SAS Proc Mixed using GC-RMA normalized data. The mean and median of each distribution were calculated using the log-fold changes.
