# Supplementary material for: Infection and genotype remodel the entire soybean transcriptome
Source: BMC Genomics. 2009 Jan 26;10:49. doi: 10.1186/1471-2164-10-49 (PMC2662884; doi:10.1186/1471-2164-10-49)
Supplement: Additional file 5 — Supplemental Figure 3. Distributions of expression changes in the Sloan upper infection court among six functional categories. The data describe the fractions of genes with different expression changes in the Sloan upper infection court within specific functional categories. [file 1471-2164-10-49-S5.pdf]

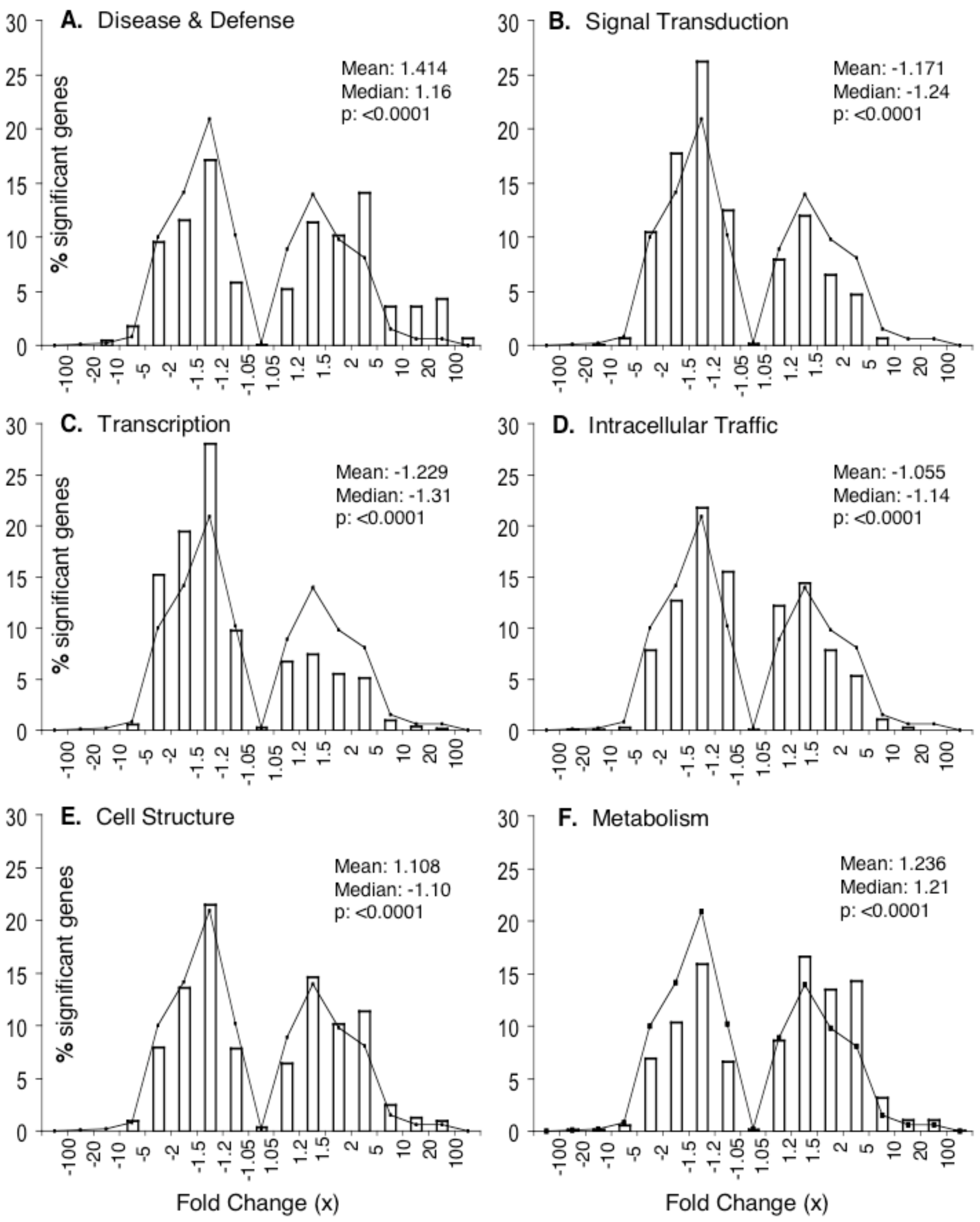

A. Disease & Defense
Mean: 1.414
Median: 1.16
p: <0.0001
B. Signal Transduction
Mean: -1.171
Median: -1.24
p: <0.0001
C. Transcription
Mean: -1.229
Median: -1.31
p: <0.0001
D. Intracellular Traffic
Mean: -1.055
Median: -1.14
p: <0.0001
E. Cell Structure
Mean: 1.108
Median: -1.10
p: <0.0001
F. Metabolism
Mean: 1.236
Median: 1.21
p: <0.0001
% significant genes
0 5 10 15 20 25 30
-100 -20 -10 -5 -2 -1.5 -1.2 -1.05 1.05 1.2 1.5 2 5 10 20 100
Fold Change (x)

Supplemental Figure 3. Distributions of expression changes in the Sloan upper infection court among six functional categories. Six functional categories relevant to infection are shown. Distributions are shown for genes showing significant infection responses (Upper vs. Mock) in Sloan as revealed by LMMA analysis of GC-RMA preprocessed microarray data with TST-FDR adjusted p value ≤ 0.01. Histograms show the number of genes in each fold change range. The line graph connects dots showing the numbers of genes in all categories in each fold change range. A negative fold change indicates a reduction in expression by that factor. p-values in each panel indicate the result of a Kolmogorov-Smirnov test comparing the distribution of expression changes within the category to the distribution for all genes. The mean and median of each distribution were calculated using the log-fold changes.
