# Supplementary material for: Infection and genotype remodel the entire soybean transcriptome
Source: BMC Genomics. 2009 Jan 26;10:49. doi: 10.1186/1471-2164-10-49 (PMC2662884; doi:10.1186/1471-2164-10-49)
Supplement: Additional file 6 — Supplemental Figure 4. Distributions of expression changes in the V71-370 lower court among six functional categories. The data describe the fractions of genes with different expression changes in the V71-370 lower court within specific functional categories. [file 1471-2164-10-49-S6.pdf]

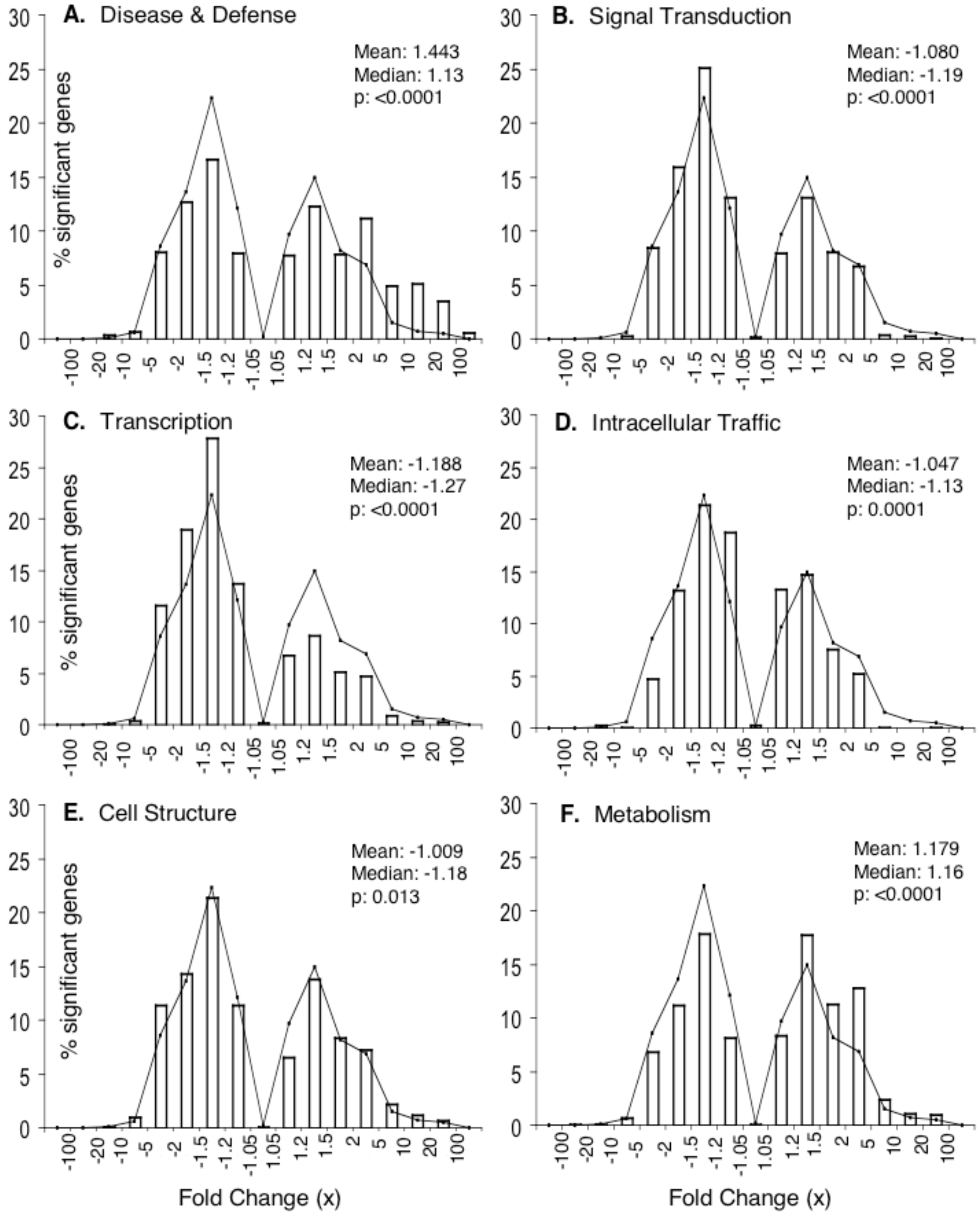

A. Disease & Defense
Mean: 1.443
Median: 1.13
p: <0.0001
B. Signal Transduction
Mean: -1.080
Median: -1.19
p: <0.0001
C. Transcription
Mean: -1.188
Median: -1.27
p: <0.0001
D. Intracellular Traffic
Mean: -1.047
Median: -1.13
p: 0.0001
E. Cell Structure
Mean: -1.009
Median: -1.18
p: 0.013
F. Metabolism
Mean: 1.179
Median: 1.16
p: <0.0001
% significant genes
0
5
10
15
20
25
30
-100
-20
-10
-5
-2
-1.5
-1.2
-1.05
1.05
1.2
1.5
2
5
10
20
100
Fold Change (x)

Supplemental Figure 4. Distributions of expression changes in the V71-370 lower court among six functional categories. Six functional categories relevant to infection are shown. Distributions are shown for genes showing significant infection responses (Lower vs. Mock) in V71-370 as revealed by LMMA analysis of GC-RMA preprocessed microarray data with TST-FDR adjusted p value ≤ 0.01. Histograms show the number of genes in each fold change range. The line graph connects dots showing the numbers of genes in all categories in each fold change range. A negative fold change indicates a reduction in expression by that factor. p-values in each panel indicate the result of a Kolmogorov-Smirnov test comparing the distribution of expression changes within the category to the distribution for all genes. The mean and median of each distribution were calculated using the log-fold changes.
