# Supplementary material for: Infection and genotype remodel the entire soybean transcriptome
Source: BMC Genomics. 2009 Jan 26;10:49. doi: 10.1186/1471-2164-10-49 (PMC2662884; doi:10.1186/1471-2164-10-49)
Supplement: Additional file 7 — Supplemental Figure 5. Distributions of expression changes in the V71-370 upper court among six functional categories. The data describe the fractions of genes with different expression changes in the V71-370 upper court within specific functional categories. [file 1471-2164-10-49-S7.pdf]

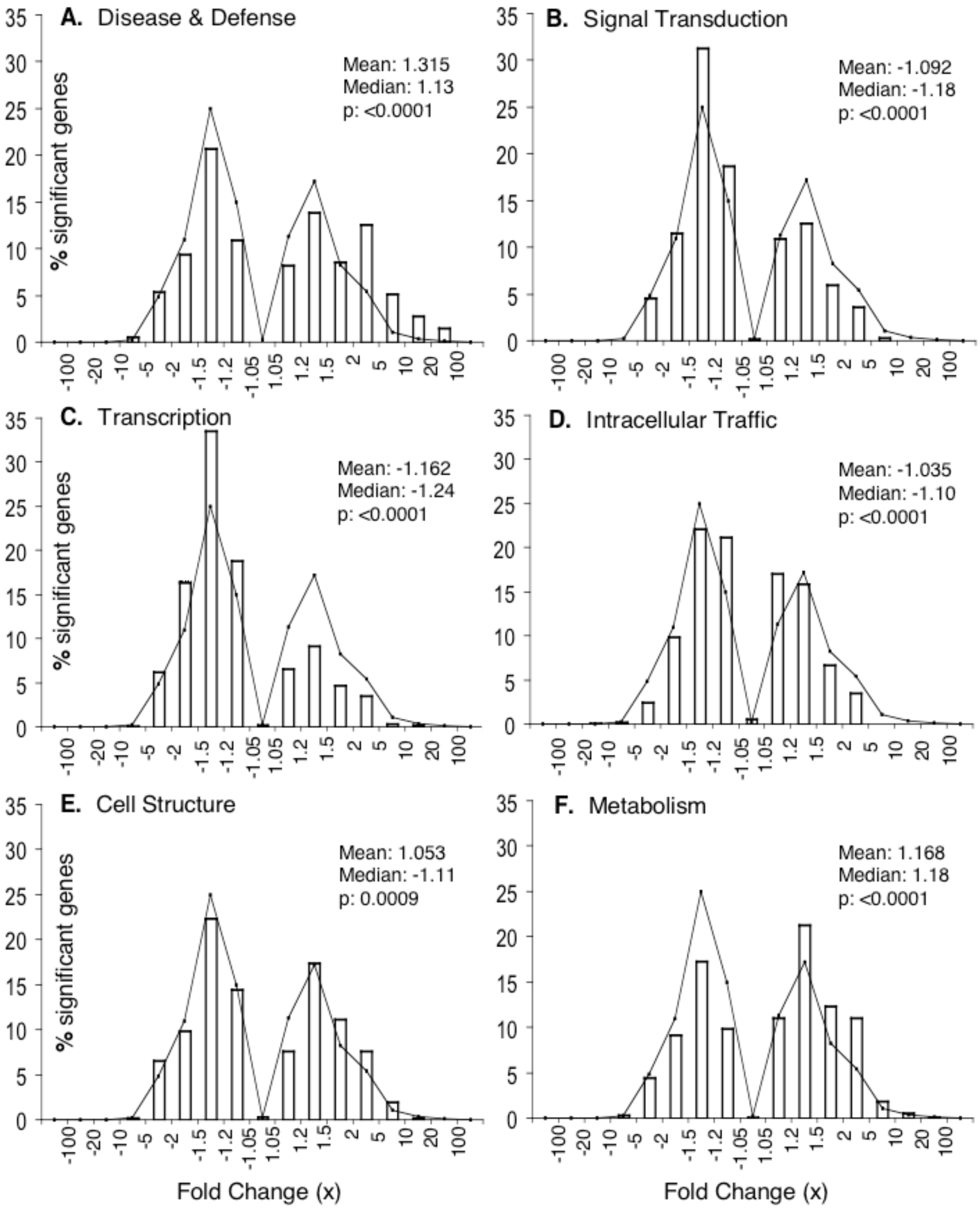

A. Disease & Defense
Mean: 1.315
Median: 1.13
p: <0.0001
B. Signal Transduction
Mean: -1.092
Median: -1.18
p: <0.0001
C. Transcription
Mean: -1.162
Median: -1.24
p: <0.0001
D. Intracellular Traffic
Mean: -1.035
Median: -1.10
p: <0.0001
E. Cell Structure
Mean: 1.053
Median: -1.11
p: 0.0009
F. Metabolism
Mean: 1.168
Median: 1.18
p: <0.0001
% significant genes
Fold Change (x)
-100 -20 -10 -5 -2 -1.5 -1.2 -1.05 1.05 1.2 1.5 2 5 10 20 100
0 5 10 15 20 25 30 35

Supplemental Figure 5. Distributions of expression changes in the V71-370 upper court among six functional categories. Six functional categories relevant to infection are shown. Distributions are shown for genes showing significant infection responses (Upper vs. Mock) in V71-370 as revealed by LMMA analysis of GC-RMA preprocessed microarray data with TST-FDR adjusted p value ≤ 0.01. Histograms show the number of genes in each fold change range. The line graph connects dots showing the numbers of genes in all categories in each fold change range. A negative fold change indicates a reduction in expression by that factor. p-values in each panel indicate the result of a Kolmogorov-Smirnov test comparing the distribution of expression changes within the category to the distribution for all genes. The mean and median of each distribution were calculated using the log-fold changes.
