# Supplementary material for: Infection and genotype remodel the entire soybean transcriptome
Source: BMC Genomics. 2009 Jan 26;10:49. doi: 10.1186/1471-2164-10-49 (PMC2662884; doi:10.1186/1471-2164-10-49)
Supplement: Additional file 8 — Supplemental Table 3. Distributions of expression differences of genes in functional categories compared with the overall differences distribution. Detailed data summarizing numbers of genes with different expression differences in six functional categories compared with the overall distribution, in two cultivars and both infection courts. [file 1471-2164-10-49-S8.pdf]

Supplemental Table 3. Distributions of expression differences of genes in functional categories compared with the overall differences distribution

Genotype[a]	Infection court[b]	Expression change range[c]	KS test[d]	Functional category[e] Disease & defense	Signal transduction	Transcription	Intracellular traffic	Cell structure	Metabolism
Sloan	Lower	Full	n1	850	1,976	2,385	815	1,097	3,337
			n2	23,556	23,556	23,556	23,556	23,556	23,556
			p	<0.0001	<0.0001	<0.0001	0.0008	0.006	<0.0001
		±2X	n1	463	1,470	1,639	640	730	2,230
			n2	16,879	16,879	16,879	16,879	16,879	16,879
			p	0.03	0.0002	<0.0001	0.002	<0.0001	<0.0001
		±1.5X	n1	269	1,006	1,064	484	442	1,420
			n2	11,200	11,200	11,200	11,200	11,200	11,200
			p	0.34	0.018	<0.0001	0.017	0.0003	<0.0001
		±1.2X	n1	92	361	377	195	120	431
			n2	3,947	3,947	3,947	3,947	3,947	3,947
			p	0.57	0.37	0.0008	0.23	0.36	0.0004
	Upper	Full	n1	823	1,878	2,267	784	1,038	3,245
			n2	22,758	22,758	22,758	22,758	22,758	22,758
			p	<0.0001	<0.0001	<0.0001	<0.0001	<0.0001	<0.0001
		±2X	n1	505	1,566	1,756	664	776	2,341
			n2	17,787	17,787	17,787	17,787	17,787	17,787
			p	0.094	<0.0001	<0.0001	0.0067	0.094	<0.0001
		±1.5X	n1	327	1,114	1,195	504	529	1,561
			n2	12,361	12,361	12,361	12,361	12,361	12,361
			p	0.21	<0.0001	<0.0001	0.029	0.088	<0.0001
		±1.2X	n1	93	389	391	219	153	501
			n2	4,414	4,414	4,414	4,414	4,414	4,414
			p	0.67	0.0058	0.0011	0.14	0.12	0.0002

Supplemental Table 3 (continued)

Genotype[a]	Infection court[b]	Expression change range[c]	KS test[d]	Functional category[e] Disease & defense	Signal transduction	Transcription	Intracellular traffic	Cell structure	Metabolism
V71-370	Lower	Full	n1	802	1,813	2,228	741	1,015	3,085
			n2	21,772	21,772	21,772	21,772	21,772	21,772
			p	<0.0001	<0.0001	<0.0001	0.0001	0.013	<0.0001
		±2X	n1	524	1,518	1,816	662	772	2,309
			n2	17,661	17,661	17,661	17,661	17,661	17,661
			p	0.18	<0.0001	<0.0001	0.0008	0.099	<0.0001
		±1.5X	n1	359	1,079	1,283	507	538	1,620
			n2	12,925	12,925	12,925	12,925	12,925	12,925
			p	0.17	0.0004	<0.0001	0.0013	0.099	<0.0001
		±1.2X	n1	124	385	476	244	191	513
			n2	4,824	4,824	4,824	4,824	4,824	4,824
			p	0.23	0.02	<0.0001	0.092	0.018	0.01
	Upper	Full	n1	736	1,651	2,044	698	914	2,904
			n2	20,418	20,418	20,418	20,418	20,418	20,418
			p	<0.0001	<0.0001	<0.0001	<0.0001	0.0009	<0.0001
		±2X	n1	530	1,511	1,831	653	764	2,365
			n2	17,960	17,960	17,960	17,960	17,960	17,960
			p	0.12	<0.0001	<0.0001	0.0007	0.01	<0.0001
		±1.5X	n1	398	1,225	1,400	538	570	1,729
			n2	14,034	14,034	14,034	14,034	14,034	14,034
			p	0.15	<0.0001	<0.0001	0.0002	0.22	<0.0001
		±1.2X	n1	143	492	529	275	205	614
			n2	5,438	5,438	5,438	5,438	5,438	5,438
			p	0.51	0.006	<0.0001	0.028	0.029	<0.0001

[a]Genotypes were cultivars Sloan and V71-370. [b]Responses in both upper infection court (Upper vs. Mock) and lower infection court (Lower vs. Mock) were evaluated relative to mock inoculated tissue. [c]The expression changes were calculated by contrast analysis with SAS Proc Mixed using GC-RMA normalized data. “Full” = full range from the greatest down-regulation to the greatest up-regulation; “±2.0X” = from down-regulated 2.0x to up-regulated 2.0x; “±1.5X” = from 1.5x down-regulated to 1.5x up-regulated; “±1.2X” = from 1.2x down-regulated to 1.2x up-regulated. [d]Kolmogorov-Smirnov test for distribution differences between a particular functional category and the whole gene set, for genes with significant infection responses: n1 = number of the significant genes in the functional category; n2 = the number of overall significant genes; p, the p-value generated by 10,000 fold resampling as described in the Methods, and adjusted for false discovery by the TST method. [e]Functional category of each gene drawn from annotation of the Affymetrix GeneChip by the Goldberg group at the University of California, Los Angeles (http://estdb.biology.ucla.edu/seed).
