# Supplementary material for: Infection and genotype remodel the entire soybean transcriptome
Source: BMC Genomics. 2009 Jan 26;10:49. doi: 10.1186/1471-2164-10-49 (PMC2662884; doi:10.1186/1471-2164-10-49)
Supplement: Additional file 9 — Supplemental Table 4. Genes and primers used for quantitative real time RT-PCR assay. The Affymetrix ID, functional annotation and primer sequences of the 22 genes used in the qRT-PCR assay. [file 1471-2164-10-49-S9.pdf]

Supplemental Table 4: Genes and primers used for quantitative real time RT-PCR assay.

**Affymetrix ID**	**Functional Annotation**	**Product Length (nt)**	**Sense Primer**	**Anti-sense Primer**
Gma.441.1.S1_at	Ubiquitin (AtRUB1)	122	AGTGTAGATGCCATATCAGAACC	CCACCAATAGTAGCCACAATTAC
Gma.16125.1.S1_s_at	Ribosomal protein S27-like protein	137	GCTTCTTTCTTACAATGGTTCTAC	GCCCTGGCACTTAACATCC
GmaAffx.90824.1.S1_s_at	26S proteasome subunit RPN2a	111	ATCGGATGTTGTTGATTTTGTTC	AATTGTCGCCTAAATGTTCCC
GmaAffx.90181.1.A1_at	Actin	90	AAGCACTTCCGATGAACGATAG	CTTGGCATCACTTAGCACCTTC
GmaAffx.60616.1.S1_at	Hcr9-OR2A	142	GTCTCTTCCAACAACTTCAGC	TCACAGAATCAGCGTACTCAG
Gma.447.1.S1_at	Defense associated acid phosphatase	92	CCGATGTACGAAAGCAGTTAC	CCCTAAATAGTCTCCCTGAATATC
GmaAffx.64261.1.A1_at	Quinone oxidoreductase	138	GGATGGCTCAAGGCAACC	GAGAGAGTATAGTGTGGGAGATAG
Gma.6640.1.S1_at	At5g66390 (secretory peroxidase)	104	TTGACCTTGTTGATCTAGTTGC	CTCGCCATTGCCACTTTG
GmaAffx.18905.1.S1_at	Cell death associated protein	101	CTTCCCATTTTCATTTATATCCAC	CGAAGCCGAGAAGTAAGC
Gma.17993.1.S1_at	P21 protein	89	TGTAATCGCTCAATGCTTCACC	CAGACGCAGCCCAGACAG
GmaAffx.75675.1.A1_at	Phenylalanine ammonia-lyase class III	101	TGTGTCAAGCAAAGATTGTTG	CATCGGCAGATTACTCCTAC
GmaAffx.2799.1.A1_at	NtPRp27-like protein	89	AGGGTTATGATGTTACTGCTC	AGTCCTCATCTGCTTGTTC
Gma.16733.2.S1_at	WRKY4 transcription factor	103	TTCAACACGGCAATCATAATGG	GAACTTCAAGCATCAGTCTTAGAG
GmaAffx.29692.1.S1_at	Chitinase (class II)	76	GCAAGAGTGAATGATCGGATTG	CCAAGTTAGGTCCAGTGTCC
Gma.16911.1.S1_at	Cytochrome P450 family protein	84	AGGTCATGTTTACGCTTCAC	TATATGATCTGTTAGCCACTATTC
GmaAffx.7209.1.A1_at	UDP-glucose 6-dehydrogenase	80	GGGAAACGGTAACGGAGAAG	CAGGCATCGGAGTTGTAGC
GmaAffx.47171.1.A1_at	Putative resistance protein	93	ACTGCCCTTGTTAGTTATGAAG	TATACAGCAATTCCATTCCATTTC
Gma.3473.1.S1_at	Small heat shock protein	95	ATGCCATTCCTTCCAAGTAGC	GGAGGAGTAGGGTGGGTTTG
GmaAffx.90984.1.A1_at	Germin-like protein	77	AGAGGACTAGGGTCATAGGC	ATGAAGGTTGTCTACTTGCTTG
GmaAffx.32612.1.S1_at	HI4’OMT	101	AAGAAATGGTTCAGTGAGGATAAG	AGCGTGTCAGATTCAGTGG
GmaAffx.90059.1.S1_at	HcrVf2 protein	108	GCAGTCTCCCTTCTCTATCAC	TCGCTCTTCTTTCACCATCC
GmaAffx.79817.1.S1_at	SGT1	82	GCTTGAGGAATATGAAACTGC	CAAAGTAGCAAACCTTGAGTTG
